# Series 1: The Use of *hsp*65- and *erm*(41)-Targeted Amplicon Sequencing in the Diagnostic Workflow for Non-Tuberculous Mycobacteria

**DOI:** 10.3390/tropicalmed10070192

**Published:** 2025-07-09

**Authors:** Tracy Lee, Adriana Cabrera, Kathleen Kolehmainen, Trevor Hird, Danielle Jorgensen, Alan O’Dwyer, Dan Fornika, Rupinder Kaur KhunKhun, Mabel Rodrigues, Natalie Prystajecky, John Tyson, Inna Sekirov, James E. A. Zlosnik

**Affiliations:** 1 British Columbia Centre for Disease Control, Public Health Laboratory, Vancouver, BC V6Z R4R, Canada; tracy.lee@bccdc.ca (T.L.); adriana.cabreradelgado@phsa.ca (A.C.); kathleen.kolehmainen@bccdc.ca (K.K.); trevor.hird@bccdc.ca (T.H.); danielle.jorgensen@bccdc.ca (D.J.); alan.odwyer@phsa.ca (A.O.); dan.fornika@bccdc.ca (D.F.); rupinder.khunkhun@bccdc.ca (R.K.K.); mabel.rodrigues@bccdc.ca (M.R.); natalie.prystajecky@bccdc.ca (N.P.); john.tyson@bccdc.ca (J.T.); jzlosnik@bccdc.ca (J.E.A.Z.); 2 Department of Pathology and Laboratory Medicine, Faculty of Medicine, University of British Columbia, Vancouver, BC V6T 1Z4, Canada

**Keywords:** non-tuberculous mycobacteria, identification, speciation, next-generation sequencing, molecular resistance

## Abstract

Evolving technologies available to clinical laboratories and laboratory-related updates to clinical guidelines both drive the need for clinical laboratories to keep their test menu updated and in line with current technological and clinical developments. Our laboratory has developed a targeted Illumina-based amplicon next-generation sequencing (NGS) assay to interrogate the *hsp*65 and *erm(*41) genes of *Mycobacterium* spp. for the purposes of providing species-level ± subspecies-level identification of *Mycobacterium* spp. organisms in clinical samples and genotypic predictions for inducible macrolide resistance (in the case of *M. abscessus* complex members). The developed assay demonstrated 100% sensitivity and specificity for *M. tuberculosis* and *M. abscessus* complex cultured organisms, 98% ID overall concordance relative to the available reference identification, and a nearly 60% “rescue” rate for primary samples that could not be identified using our previous method. There was 94.6% concordance between genotypic and phenotypic results for inducible macrolide resistance. The developed assay was successfully implemented in our clinical laboratory and has been accredited for clinical use.

## 1. Introduction

The *Mycobacterium* genus comprises several groups, including the heterogeneous group of non-tuberculous mycobacteria (NTM). NTM are organisms with an environmental reservoir that can be isolated in the clinical laboratory as either an etiological agent of clinically significant infections, a colonizer (in particular in respiratory samples), or an environmental contaminant. They are more likely to be of clinical significance in immunocompromised patients, those with organ damage conducive to infection (e.g., cystic fibrosis or bronchiectasis patients), or post-procedure wounds [1,2,3]. The clinical significance of NTM in recent decades has been increasing, with the expanding use of immunosuppressive medications/transplantation and longer lifespans of patients with congenital immunosuppressive and other genetic conditions. It has been noted that many developed countries now have a higher incidence of NTM vs. *M. tuberculosis* infection [4,5]. As such, it is crucial for a clinical laboratory to be able to accurately and rapidly identify NTM in clinical specimens to facilitate timely and accurate clinical management where necessary. Moreover, while antimicrobial susceptibility testing is not routinely recommended for all NTM isolated in a clinical laboratory, recent clinical guidelines do recommend that baseline susceptibility testing be performed for some clinically relevant species—for instance, testing for inducible macrolide resistance in *M. abscessus* subspecies, either phenotypically via a prolonged incubation (14 days) or sequencing of the *erm*(41) gene [6].

Inducible macrolide resistance in *M. abscessus* complex (subspecies *abscessus*, *bolletii,* and *massiliense*) is conferred by a functional *erm*(41) gene, which is typically present in *M. abscessus* subsp. *abscessus*, but not in *M. abscessus* subsp. *massiliense* organisms [7]. A wild-type *erm*(41) gene produces a functional 23S rRNA methylase, and its expression is induced by exposure to macrolide antibiotics. Macrolides have a lower binding affinity to methylated 23S rRNA, resulting in inducible macrolide resistance [8]. When the *erm*(41) gene is rendered non-functional, this can be via a 276 bp deletion within the gene (truncation mutation) or loss of function of the *erm*(41) gene due to a T28C point mutation leading to an amino acid change from Trp to Arg [9,10].

The identification of NTM at the species level can be performed using various methods. Commercially available AccuProbe tests (Hologic) have been a commonly used method of *Mycobacterium avium* complex (MAC) and *M. gordonae* species identification in many clinical laboratories. However, the company discontinued the production of these products in late 2022, forcing clinical laboratories to explore other options for NTM identification. Mass spectrometry methods (MALDI-ToF), targeted PCRs, and sequencing of key housekeeping genes (e.g., 16S rRNA and *hsp*65) have also been used [11,12]. Each methodology has its pros and cons—for instance, while MALDI-ToF-based identification has been improving with expanded NTM databases, it still requires a pure culture of an organism. For the slowly growing NTM species, this requirement means prolonged delays with identification, as growing a sufficient amount of organism in pure culture might take over a week from the initial growth of the organism from a clinical specimen. Targeted PCRs usually target only a single or a small subset of species—usually at a higher cost than the previously available AccuProbe reagents. The choice of housekeeping gene for sequencing-based NTM identification needs to be carefully considered. For instance, while there are CLSI-based guidelines available for the interpretation of 16S rRNA-based species identification [13], some key clinically significant NTM complex members have indistinguishable 16S rRNA sequences (e.g., the *M. abscessus* complex group), making it an unsuitable target for this purpose. Several other essential genes have also been previously evaluated with respect to their capacity to differentiate *Mycobacterium* spp. Combinations of housekeeping genes (e.g., *gyr*B, 16S, and ITS fragment sequences [14]; *rpo*B, *arg*H, and *cya* fragment sequences [15]; or *hsp*65, *rpo*B, and ITS fragment sequences [16]), *rpo*B sequencing alone [17], and *gyr*B-targeted microarrays [14,18] have been demonstrated to have good capacity for resolving NTM organisms. Regardless of the choice of a housekeeping gene used for NTM identification, there must be careful consideration of the selection and curation of a database against which to compare the sequences. In our laboratory, we have previously developed an *hsp*65-based sequencing method for the identification of NTM species, including a curated database built for this purpose [19]. The original method was developed using Sanger sequencing, which was a well-supported technology in our laboratory. However, in recent years, the manufacturer has discontinued technical support for some of their instruments, creating a lack of contingency for our routine laboratory operations and prompting an evaluation of other sequencing platforms. With the decreasing costs and increasing use of next-generation sequencing (NGS) technologies, we have worked to develop and implement an NGS-based method to include the concurrent sequencing of a 401 bp fragment of the *hsp*65 gene (similar to our original methodology), as well as introduced an additional *erm*(41) target to evaluate inducible macrolide resistance. We describe herein this newly developed methodology.

## 2. Materials and Methods

### 2.1. Samples

**Figure 1:**
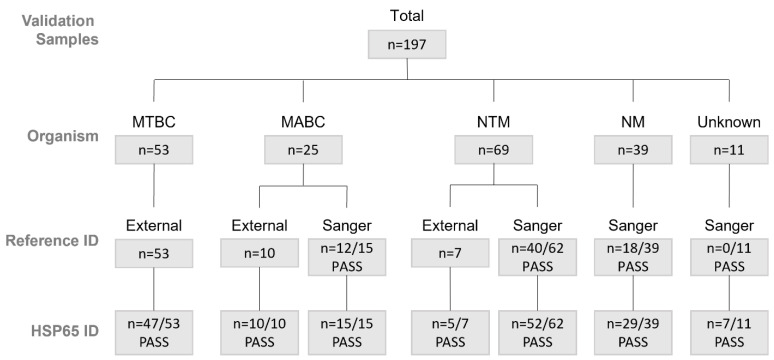
Samples used for validation. External reference ID refers to a lab-validated test, while Sanger reference ID refers to in-lab Sanger sequencing. Definition of PASS for Sanger sequencing is a 401 bp amplicon and ≥97.5% identity. Definition of PASS for *HSP*65 speciation is a 401 bp amplicon and ≥97.5% identity. MTBC = *Mycobacterium tuberculosis* complex; MABC = *Mycobacterium abscessus* complex; NTM = non-tuberculous *Mycobacterium*; NM = non-mycobacterium.

There was a total of 197 samples available for validation, including 53 *Mycobacterium tuberculosis* complex (MTBC) samples, 25 *Mycobacterium abscessus* complex (MABC) samples, 69 non-tuberculous *Mycobacterium* spp. (NTM) samples, 39 non-*Mycobacterium* spp. (NM) samples, and 11 “unknown” samples (no species identification due to previously failed Sanger sequencing) (Figure 1, Supplementary Tables S1–S5). Of these samples, 19 were direct patient samples, including sputum (12), tissue (3), pleural fluid (1), and bronchial wash or bronchoalveolar lavage (3) (see Supplementary Table S6 for additional information on direct samples used for validation). An additional 468 post-implementation clinical samples were collected between October 2023 and January 2024. These included 18 MTBC, 65 MABC, 308 NTM, 58 NM, and 19 samples with unknown identification (due to failed *hsp*65 sequencing) (Table 1, Supplementary Tables S7–S11). Of these samples, 22 were direct patient samples, including sputum (6), bronchial wash (1), tissue (5), cerebrospinal fluid (1), gastric aspirate (1), formalin-fixed paraffin-embedded (FFPE) scrolls (information on source tissue was not available) (4), and proficiency program samples designed to mimic sputum (2) (see Supplementary Table S12 for additional information on direct samples evaluated post-implementation). Clinical information on the patients whose samples were included in the evaluation was not available to the laboratory staff.

### 2.2. Sample Extraction

All primary patient samples and organism cultures were extracted according to the manufacturer’s protocol using the MagMAX™ Total Nucleic Acid Isolation Kit (Cat# AM1840) on MagMAX™ Express-96/KingFisher™ Flex automated extraction systems (ThermoFisher Scientific, Waltham, MA, USA).

### 2.3. Target Amplification for NGS

All primers were based on previously published designs and modified for this assay (Table 2). M13 universal sequencing tails were added to primers targeting. Primers were pooled and added at 2.5 μL per pool to a 25 μL reaction containing 5 μL lysate, 12.5 μL Platinum™ SuperFi II PCT Master Mix (Cat# 12368010), and 2.5 μL UltraPure™ DNase/RNase-Free Distilled Water (Cat# 10977105, ThermoFisher Scientific, Waltham, MA, USA). Amplification was completed on SimpliAmp^TM^ Thermal Cyclers (ThermoFisher Scientific, Waltham, MA, USA) using the following conditions: 1 cycle at 98 °C for 30 s, followed by 35 cycles at 98 °C for 10 s, 60 °C for 10 s, and 72 °C for 30 s, and a final extension at 72 °C for 5 min.

### 2.4. Library Preparation and Sequencing by NGS

All samples were prepared for sequencing using a modified version of the manufacturer’s protocol for the Illumina DNA Prep, (M) Tagmentation, 96 Sample Library Prep Kit (Cat# 20018705, Illumina, San Diego, CA, USA), which was developed and validated at the BC Center for Disease Control Public Health Laboratories (BCCDC PHL) [21]. Libraries were quantified using a Qubit™ dsDNA Quantitation assay kit (Cat# Q32851) and Qubit 3 Fluorometer (ThermoFisher Scientific, Waltham, MA, USA) and then sequenced using the Illumina sequencing platform and 300-cycle kits (Illumina, San Diego, CA, USA).

### 2.5. Bioinformatics

The bioinformatics pipeline (https://github.com/BCCDC-PHL/AMR-TB-amplicon-artic-nf [v0.2.2] (accessed on 2 July 2025); referred to as the NGS pipeline throughout this manuscript) was based on the BCCDC-PHL/ncov2019-artic-nf pipeline (https://github.com/BCCDC-PHL/ncov2019-artic-nf (accessed on 2 July 2025)), which was a fork of the https://github.com/connor-lab/ncov2019-artic-nf (accessed on 2 July 2025) pipeline [22] and was adapted to support the analysis of *Mycobacterium* species by targeted NGS. Briefly, the NGS pipeline builds a *hsp*65 consensus sequence from Illumina paired-end reads, which is then subjected to comparison against an in-house curated *hsp*65 database (v.15.4) using BLASTn. This database was originally established by McNabb et al. [19]. Quality control (QC) checks were implemented to evaluate the quality of the assembly and taxonomic assignment. For the analysis, the definition of “PASS” criteria for *hsp*65 speciation was defined as samples with a 401 bp amplicon size and matches exhibiting ≥97.5% sequence identity by BLASTn. Samples with 401 bp amplicon size but <97.5% sequence identity were flagged for “REVIEW” and those with amplicon size <401 bp were flagged as “FAIL/REPEAT”. The NGS pipeline flags whether a sample may be polymicrobial (“MIXED_BASES”) by indicating the detection of ambiguous bases in the *hsp*65 amplicon. Samples identified as *M. abscesses* complex (MABC) subspecies (*abscessus*, *bolletii,* and *massiliense*) were assessed for the presence of a 276 bp deletion in the *erm*(41) gene, which is reported by the NGS pipeline as “susceptible”, whereas full-length/intact *erm*(41) is reported as “resistant”. Additionally, the NGS pipeline reports other mutations if detected (e.g., T28C). The detection of T28C in full-length *erm*(41) was predicted as “susceptible”, as this point mutation has shown a change in inducible resistance by others [23,24]. For this analysis, the definition of “PASS” criteria for the *erm*(41) amplicon was ≥90% coverage relative to the reference sequence (NCBI CU458896.1 for full-length *erm*(41) and NCBI AP014547.1 for truncated *erm*(41)). Samples with coverage < 90% were flagged as “REVIEW/REPEAT”. All validation analyses were completed using version 0.2.2 of this pipeline.

### 2.6. Reference Methods

For accuracy assessment, *hsp*65-based speciation by the NGS pipeline was compared to *hsp*65 Sanger sequencing [19] or another validated assay used by our laboratory. The definition of “PASS” for Sanger sequencing is also a 401 bp amplicon with ≥97.5% sequence identity match (determined using broth microdilution with prolonged incubation for up to 14 days, performed at the National Microbiology Laboratory (NML) according to established clinically recommended standards [25]).

### 2.7. Validation of hsp*65* Speciation

The validation parameters assessed for *hsp*65-based speciation include accuracy, analytical specificity, limit of detection (LOD), and precision. Only samples with an ID provided by Sanger sequencing (“PASS”) or a previously available reference ID through another validated in-lab assay were used for validation studies. Of the 140 samples with a reference ID, 132 samples also met QC “PASS” metrics for *hsp*65 sequencing and could be used to calculate accuracy, analytical specificity, and analytical sensitivity (Figure 1). Accuracy, specificity, and sensitivity were assessed for MTBC and MABC using 2 × 2 contingency tables. Accuracy was also assessed for each organism type (e.g., MTBC, MABC, NTM, and NM) by concordance between the *hsp*65 ID and reference ID. LOD and precision were evaluated using MTBC H37Rv culture (ATCC, Manassas, VA, USA) serially diluted from 10^−2^ to 10^−7^. Briefly, pure H37Rv culture was harvested from solid Lowenstein Jennings slants into 500 μL of 0.5× TBE buffer and heat-killed at 95 °C for 10 min. Serial dilutions were made from this culture lysate in 1× IDTE buffer and sequenced in triplicate over 1–3 runs. Replicate samples were assessed for concordance of *hsp*65 ID to evaluate precision. LOD was determined relative to *M. tuberculosis* organism, as our laboratory employs a laboratory-developed (LDT) targeted PCR for *M. tuberculosis* detection, which could be used as a convenient reference point since one of the PCR targets is the single-copy *mpt*64 gene. LOD was determined to be the lowest dilution where all replicates were successfully sequenced; the LOD was quantified relative to *mpt*64 Ct values obtained on the LDT PCR assay. Briefly, the *M. tuberculosis* sample extract was inoculated directly into 1X TaqMan^®^ GTXpress™ Master Mix (CAT# 4401892, ThermoFisher Scientific, Waltham, MA, USA) with 1X primer/probe mixes targeting the *mpt*64 gene. This was completed on the Applied Bio-Systems StepOne™ Real-Time PCR System (ThermoFisher Scientific, Waltham, MA, USA) using the following cycling conditions: 1 cycle at 50 °C for 2 min and 95 °C for 30 min, then 40 cycles at 95 °C for 3 s followed by 60 °C for 20 s. LOD was also assessed on ten primary patient samples by comparing *mpt*64 Ct values to *hsp*65 QC status and across all samples with reference ID to determine minimum aligned reads and depth.

Analysis of the phylogenetic distribution of *Mycobacterium avium* complex members isolated in clinical samples pre- and post-amplicon NGS implementation was performed by extracting cultured NTM identification results from laboratory records for May 2022 to October 2023 (18 months pre-implementation; majority of *M. avium* complex members identified by Hologic (Marlborough, MA, USA) AccuProbe to complex level resolution) and November 2024 to April 2025 (18 months post-implementation; all M. *avium* complex members identified by *hsp*65 NGS to species level resolution) and comparing the datasets with respect to species identification. *M. avium* complex members were selected for comparison as an NTM organism with potential clinical significance the level of identification of which was the most impacted by methodology transition.

### 2.8. Statistics

Two-sided 95% confidence intervals were calculated for accuracy, analytical specificity, and analytical sensitivity using the Wilson score method as outlined in EP12 and Westgard QC [26,27].

### 2.9. hsp*65* Phylogeny Visualization

The *hsp*65 amplicon sequences were aligned using MAFFT (v7.520). The resulting multiple sequence alignment was used as input for IQ-TREE (2.4.0) for maximum likelihood (ML) phylogenetic tree inference using ModelFinder Plus (-m MFP) (from https://iqtree.github.io/ (accessed on 2 July 2025) to determine the best-fit model. Branch support was assessed using 1000 ultrafast bootstrap replicates. The resulting ML tree was visualized using iTOL (Interactive Tree of Life, https://itol.embl.de/ (accessed on 2 July 2025)).

## 3. Results

### 3.1. Accuracy of hsp*65* Speciation

Accuracy was assessed on 132 samples that had a reference ID provided and passed *hsp*65 sequencing QC criteria. For MTBC, a true positive (TP) was defined as both *hsp*65 and reference IDs belonging to MTBC species, a true negative (TN) as neither belonging to MTBC species (e.g., MABC, NTM, NM, or non-MTBC species), a false positive (FP) as *hsp*65 providing an MTBC ID when the reference ID was of a different species, and a false negative (FN) as *hsp*65 providing a different species ID when the reference identified the sample as belonging to MTBC. MTBC includes *M. tuberculosis, M. caprae,* and *M. bovis*, which cannot be distinguished by *hsp*65 sequencing (Supplementary Figure S1). Similar definitions were used for MABC, which includes the subspecies *abscessus, bolletti*, and *massiliense.* MTBC and MABC had calculated accuracies of 100% with 95% confidence intervals of 97.2–100% (Table 3 and Table 4). A concordance calculation was also completed for each organism type and gave values of 100% for MTBC, MABC, and NM and 93% for NTM (Table 5). The lower NTM concordance was due to two species discrepancies between the reference and the *hsp*65 ID: *M. gordonae* that was identified by *hsp*65 as *M. neoaurum* (SPEC-48), and *M. gordonae* that was identified by *hsp*65 as *M. avium* (SPEC-50) (Supplementary Table S3). Both SPEC-48 and SPEC-50 had high proportions of non-*Mycobacterium* reads, with only 2% of SPEC-48 reads and 0.91% of SPEC-50 reads being assigned as the *Mycobacterium* genus and at least 93% being detected as *Homo sapiens* in each sample. SPEC-50 was also confirmed as a mixed infection, as the sample came from a patient who had a history of cultures with mixes of *M. gordonae* and *M. avium*. At the time of this validation, the NGS pipeline only reported the highest mapped ID, and as such could not detect mixed infections. It has since been updated to report the top three mapped IDs, allowing for the resolution of mixed infections (https://github.com/BCCDC-PHL/AMR-TB-amplicon-artic-nf [v.0.3.4] (accessed on 2 July 2025)). Note that although SPEC-116 showed a discrepancy between the reference (*M. paraintracellulare*) and *hsp*65 ID (*M. intracellulare*), the reference sequence in the database, which was originally thought to be *M. paraintracellulare*, was later confirmed to be *M. intracellulare,* suggesting that this sample is actually concordant. Overall, the concordance of the assay was 98% (Table 5).

### 3.2. Analytical Specificity and Sensitivity of hsp*65* Speciation

Analytical specificity and sensitivity were assessed on 132 samples that had a reference ID provided and passed *hsp*65 sequencing QC criteria. MTBC samples included *M. tuberculosis* and *M. bovis* (Supplementary Table S1)*,* while MABC samples included subsp. *abscessus*, *massiliense*, and *bolletti* (Supplementary Table S2). NTM organisms (excluding MABC) included the *Mycobacterium* species *poriferae*, *avium*, *timonense*, *chelonae*, *haemophilum*, *gordonae*, *brisbanense*, *xenopi*, *phocaicum*, *branderi*, *peregrinum*, *asiaticum*, *kumamotonense*, *paraintracellulare*, *paragordonae*, *cosmeticum*, *septicum*, *fortuitum*, *alsense*, *pulveris*, *rutilum*, *novomagense*, *neuaurum*, and *brumae* (Supplementary Table S3). Non-*Mycobacterium* organisms included the acid-fast and weakly acid-fast bacteria *Tsukamurella*, *Nocardia*, *and Gordonia* and the non-acid-fast *Actinomadura* (Supplementary Table S4). *Rhodococcus* (a weakly acid-fast bacterium) was also tested, but the sample used failed both Sanger and Illumina *hsp*65 sequencing. Analytic specificity and sensitivity were calculated for MTBC and MABC. MTBC had a specificity of 100% (95% CI: 95.7–100%) and a sensitivity of 100% (95% CI: 92.4–100%). MABC had a specificity of 100% (95% CI: 96.6–100%) and a sensitivity of 100% (95% CI: 85.1–100%).

### 3.3. Limit of Detection (LOD) of hsp*65* Speciation

**Figure 2:**
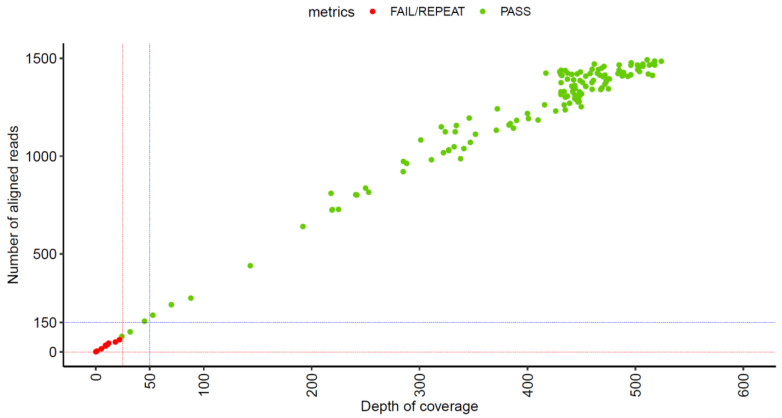
Number of reads and depth of coverage for *hsp*65 amplicon. Each sample is represented by a dot in the graph. Bioinformatics QC metrics check flagged by the pipeline are colored with red if ‘FAIL/REPEAT’ or green if ‘PASS’. A total of 140 samples were included. Red dashed lines indicate the region where the samples ‘fail’ QC metrics. Blue dashed lines indicate new threshold quality filter criteria to improve the current quality filter.

For the wet lab component, the LOD was determined to be *mpt*64 Ct 35.81 (10^−5^ dilution), which was the lowest dilution at which all H37Rv replicates (9/9) were detected with a full-length amplicon (401 bp) and 100% sequence identity match to *M. tuberculosis* (Table 6, Supplementary Table S13). LOD was also evaluated in direct samples. The lowest sample that still passed QC had an *mpt*64 Ct value of 35.26, corresponding to a smear result of 1 + (>10) (Table 7), and which is in line with H37Rv result. For the bioinformatics component, 140 samples were assessed for minimum depth of coverage and number of aligned reads. As shown in Figure 2, samples with a depth of coverage of ~25x (even though some of these were concordant with the reference ID) failed to pass the QC criteria and were flagged by the pipeline as ‘FAIL/REPEAT’. This suggests that >25x coverage is required for full-length amplicon size (401 bp), ≥97.5% identity, and accuracy. Based on these results, a depth of 50x will be incorporated in the current bioinformatics QC metrics check criteria with the aim of improving the quality filter. This depth of coverage is associated with a minimum of 150 aligned reads.

### 3.4. Precision of hsp*65* Speciation

Sequencing of *hsp*65 in H37Rv replicates gave a correct *M. tuberculosis* ID across all replicates at culture dilutions of 10^−1^ down to 10^−5^ (Table 6, Supplementary Table S13).

### 3.5. Failure Rate of hsp*65* Speciation

Of the 140 samples with a reference ID (provided through one of our clinical laboratory’s validated tests), 9 failed Illumina *hsp*65 sequencing (given a flag of FAIL/REPEAT, REVIEW, or MIXED_BASES). These included six MTBC, two NTM, and one NM sample, giving an overall *hsp*65 Illumina sequencing failure rate of 6.4% (Table 8). Samples that failed both Illumina and Sanger *hsp*65 sequencing were not included in this calculation, since they were not considered to have a well-characterized reference ID. Note that the one NM sample (SPEC-84) passed Sanger sequencing but was flagged as MIXED_BASES by Illumina *hsp*65 sequencing; however, the pipeline still provided an ID that was concordant with that from Sanger sequencing (Supplementary Table S4).

### 3.6. Rescue Rate of hsp*65* Speciation

There were 127 samples on which Sanger sequencing was previously performed; of these, 57 failed Sanger sequencing (3 MABC, 22 NTM, 21 NM, and 11 unknown). The Illumina *hsp*65 sequencing was successful on 34 of these samples, giving an overall rescue rate of 59.6% (Table 8). These included all MABC samples. Note that there were an additional eight samples (four NTM, one NM, and three unknown) that had an Illumina *hps*65 sequence result but were flagged as either “MIXED_BASES” or “REVIEW” (rather than “FAIL/REPEAT”) (Supplementary Tables S3–S5). For samples that failed both Illumina NGS and Sanger sequencing (n = 23), it is possible that these samples may have been of poor quality and unsuitable for sequencing.

### 3.7. Post-Implementation Clinical Samples Assessed by hsp*65* Sequencing

Following validation, 468 clinical samples (both cultures and primary patient samples) were collected between October 2023 and January 2024. The Illumina NGS *hsp*65 assay successfully sequenced 17/18 MTBC samples, 62/65 MABC samples, 307/308 NTM samples (excluding MABC), and 49/58 NM samples (Table 1). All samples in these groups that did not “PASS” sequencing could still be speciated by the NGS pipeline (Supplementary Tables S7–S10). Only 11/468 samples could not be speciated and these also had few (<12) to no aligned reads, suggesting poor-quality samples (Supplementary Table S11). Note that this was the first time FFPE scrolls were tested, and 1/4 had an organism successfully identified by both MPT64 qPCR and *hsp*65 sequencing.

For phylogenetic distribution comparison of MAC clinical isolates, 1253 MAC members were identified in 18 months pre-NGS implementation and 1157 MAC members were identified in 18 months post-NGS implementation. Transition to *hsp*65 NGS-based identification uncovered significant representation of *M. timonense*, *M. intracellulare*, and *M. chimaera* species, which previously would have been identified as MAC members only (Table 9).

**Figure 3:**
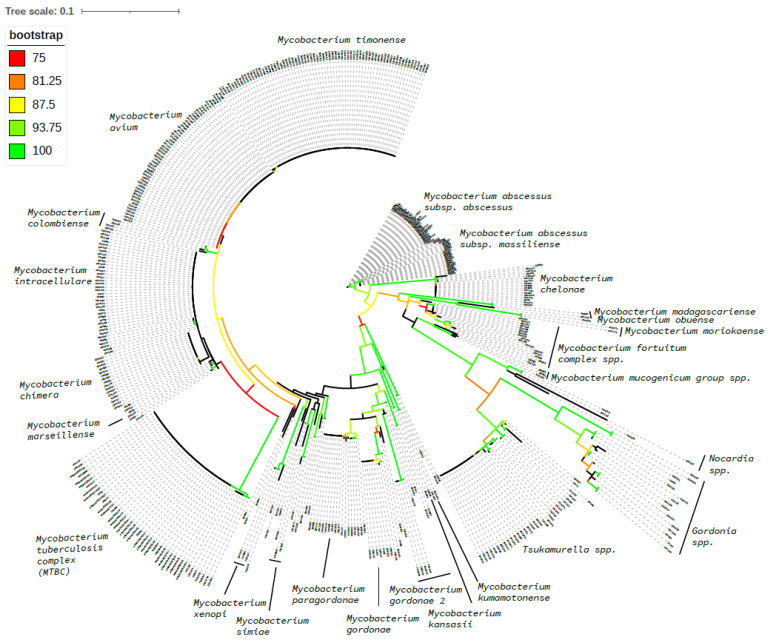
Phylogenetic tree constructed using IQ-TREE based on *hsp*65 sequence alignment. The tree was inferred using the maximum likelihood method and 1000 ultrafast bootstrap replicates. Visualization was performed in iTOL (https://itol.embl.de/ (accessed on 2 July 2025)) using the circular layout, with branch colors indicating bootstrap support values.

A subset of *hsp*65 sequences generated during the method validation and the first three months post-implementation are visualized in a phylogenetic tree (Figure 3), demonstrating the lower level of relatedness between *Mycobacterium* spp. and non-*Mycobacterium* spp. groups and much higher relatedness within some *Mycobacterium* spp. complexes, such as MAC and MABC.

### 3.8. Mycobacterium Abscessus erm*(41)* Amplicon and Macrolide Resistance

After *hsp*65 speciation, the NGS pipeline filters the samples identified as MABC subspecies (*abscessus*, *bolletii,* and *massiliense*) to assess for a truncated *erm*(41) gene, a 276 bp deletion that results in a non-functional *erm*(41), which prevents the induction of macrolide resistance [23,24]. This included 29 validation samples and 65 clinical samples that passed QC metrics for *erm(*41) amplicon sequencing (Supplementary Tables S14 and S15). The NGS pipeline is also able to identify all mutations in the *erm*(41) amplicon, as some have been associated with a lack of inducible macrolide resistance. For example, T28C is known to inactivate the 23S RNA methylase, preventing the induction of resistance to macrolides [10]. There were 6 validation samples and 31 clinical samples with phenotypic macrolide susceptibility testing performed. Of these, 35/37 were concordant based on resistance/susceptibility prediction using *erm*(41) amplicon size (truncated vs. full-length/intact) and the T28C mutation known to confer susceptibility, giving a concordance of 94.6% (Table 10, Supplementary Table S15).

We did observe two discrepant isolates, 22A616 and 22H476, which lacked markers of inducible macrolide resistance yet showed inducible resistance phenotypically. Isolate 22A616 tested susceptible to macrolide without prolonged incubation, indicating that the discrepancy was not likely to be related to the acquisition of mutations in other macrolide resistance-associated genes. Isolate 22H476 tested intermediate to macrolides without prolonged incubation.

## 4. Discussion

The increased clinical significance of NTM warrants clinical laboratories to have on menu rapid and reliable methods for their differentiation from *M. tuberculosis* and species/subspecies-level identification. This is particularly true for clinical laboratories in developed countries, where the incidence of NTM disease can be on par with or higher than the incidence of *M. tuberculosis* disease. Similarly, with recent recommendations from IDSA guidelines to assess baseline susceptibilities for clinically relevant cases [6], it is prudent to develop methodologies that can provide preliminary genotypic-based predictions of susceptibilities for these relatively slow-growing organisms.

NGS technologies are continuously evolving, providing laboratories with increasing efficiencies, often at lower costs. This becomes particularly attractive when previously available technologies become outdated or commercially available products are discontinued. Our laboratory successfully introduced an Illumina NGS amplicon sequencing assay that combines NTM identification with inducible macrolide resistance prediction for *M. abscessus* complex members and has been accredited by both the Diagnostic Accreditation Program (DAP—our jurisdiction’s regulatory body) and the College of American Pathologists (CAP). We chose Illumina as our sequencing platform, based on it being the most utilized technology in our laboratory, to facilitate multiplexing of different organisms, increasing efficiencies. Depending on individual laboratory preferences, other sequencing platforms, e.g., Nanopore, another platform commonly available in clinical microbiology laboratories, can be chosen for targeted NGS for rapid organism identification and resistance testing [28]. While Illumina allows for an efficient multiplexing of larger runs, Nanopore would have the potential to offer faster turn-around times for individual samples, though potentially at higher costs. Metagenomics sequencing has the potential to offer even higher resolution and a reliable identification of polymicrobial infections [29]. However, metagenomics approaches are particularly costly, which would be prohibitive for routine use in most laboratories. For our laboratory, the cost per sample of this amplicon NGS assay is only CAD 17 higher than the combined cost of previously utilized Sanger *hsp*65 sequencing and AccuProbe testing, which was our previous NTM identification workflow. The amplicon NGS assay, however, offers both identification and inducible macrolide resistance prediction. The decreased hands-on time required for NGS-based testing versus the previously utilized Sanger testing allowed us to increase the frequency of *hsp*65 amplicon sequencing to twice weekly, improving turn-around times and thus optimizing clinical utility. These findings are concordant with recently published assessments of various molecular technologies available for NTM identification [12]. Our observed discrepancies between the *hsp*65 organism ID on direct sample sequencing vs. the organism ultimately isolated in culture, for cases that had documented instances of coinfection with multiple organisms, highlight the need to evaluate for multiple coinfections whenever possible. Additionally, this brings up the possibility of the differential growth of one organism vs. another depending on the clinical context, e.g., concurrent antimicrobial pressures or competitive inhibition from other members of normal respiratory flora. The two organisms that had discrepant genotypic/phenotypic results for inducible macrolide resistance highlight that while the *erm*(41) gene has very high predictive potential for inducible macrolide resistance phenotype, phenotypic confirmation remains important. Point mutations in *erm*(41) have been described in association with the duration of time to inducible resistance [30], and perhaps future studies will uncover additional less common genetic loci that increase the reliability of the prediction of this phenotype.

Our assay has undergone optimization over the course of its development and implementation, with the bioinformatics pipeline initially capturing only the top ID choice for the *hsp*65 sequence and currently optimized to report the top three choices to facilitate the detection of potential mixed infections. Possible future optimizations can be considered, such as incorporating additional target genes for identification purposes, or broader antimicrobial predictions. With both the current design and any future modifications, carefully setting up quality parameters for both the wet lab and bioinformatics portions of the test, as well as using well-curated databases for sequence comparisons, are crucial components of a high-quality clinical sequencing program that would meet the stringent requirements of clinical laboratory accreditation bodies.

Since the introduction of amplicon NGS testing as the primary identification method for NTM in our laboratory, one interesting finding that was observed was that the increased resolution of NTM ID can at times result in increased difficulties for clinical decision making. Previously, the majority of *M. avium* complex (MAC) isolates were identified via the Hologic AccuProbe at the complex level only. The current approach provides their ID at the individual species level, and this complex has multiple species assigned to it, with ongoing proposed taxonomic rearrangements [31,32]. Individual complex members can have highly related *hsp*65 sequences, with sometimes just one nucleotide difference between type species; this high level of similarity can be observed in the phylogenetic tree generated from a subset of our *hsp*65 sequences (Figure 3). Our methodological transition has uncovered interesting insights into the phylogenetic distribution of clinical isolates from this group of organisms (Table 9), which in the future can be coupled with clinical information to assess their relative clinical significance as causative agents of disease. In particular, we have seen a significant number of *M. timonense* isolated in our laboratory, despite the fact that this organism has only very rarely been reported in the literature in association with clinically significant infections [33,34,35]. In addition, since transitioning to our amplicon NGS assay, we have witnessed ID results for organisms isolated from samples of the same patient collected over various periods of time identified as different MAC species members, whereas previously, they would have all been identified as “*Mycobacterium avium* complex”. In some cases, this has resulted in clinical conundrums (per consultations received from treating physicians), with clinicians struggling to determine the significance of the isolates and whether what is being observed is a persistent infection or multiple reinfection episodes. Additional genotyping, potentially by whole-genome sequencing, might be necessary to help determine the true relatedness and significance of NTM organisms in this type of situation, and future studies examining this phenomenon might shed light on this clinical conundrum and potentially result in updates to clinical guidelines. The true and full extent of these methodological adaptations on clinical decision making and outcomes is not currently known, as NTM are not a reportable organism in our jurisdiction, making systematic data collection difficult. Future studies, however, can focus on a systematic review of clinical presentations, treatment approaches, and outcomes to gain better insights into the clinical significance of different NTM organisms and their persistence, reinfection, or coinfection. The current literature offers sparse guidance for the clinical management of cases of NTM relapse vs. NTM reinfection, although at least one recent example highlighted that different clinical courses can be entertained (e.g., prolongation of treatment course in cases of relapse [36]). Relapse vs. reinfection outcomes of NTM treatment are reported separately in the literature [37,38], and current international NTM practice guidelines do recommend comparing consecutive patient isolates to determine their relatedness [6]. However, definitive clinical guidance on approaches to patients, either post-relapse or post-reinfection, has not been published to our knowledge and, variability in methods to classify relapse vs. reinfection cases also further complicates the situation.

Overall, our laboratory has had a very positive experience with transitioning to amplicon NGS testing for NTM identification and inducible macrolide resistance predictions, from both the clinical utility and workflow perspectives. While careful attention to quality metrics is paramount with such transitions, and they have the potential to surface new questions regarding clinical significance interpretations, NGS technologies continue to prove themselves to be tremendously helpful in a microbiological clinical laboratory.

## Figures and Tables

**Table 1:** Overview of clinical samples sequenced post-implementation. Samples were IDed through *hsp*65 speciation. Those that are “unknown” could not be previously IDed in our laboratory due to failed Sanger sequencing. MTBC = *Mycobacterium tuberculosis* complex; MABC = *Mycobacterium abcesses* complex; NTM = non-tuberculous *Mycobacterium*; NM = non-mycobacterium; PASS NGS = passed NGS-based identification.

Sample ID	Total Samples	Direct	Culture	PASS NGS
MTBC	18	9	9	17
MABC	65	2	63	62
NTM	308	3	305	270
NM	58	0	58	49
Unknown	19	8	11	0
Total	468	22	446	398

**Table 2:** Primer sequences. Universal M13 tails in *hsp*65 are underlined. For *erm*(41) amplicon size, R = resistant; S = susceptible. Conc. = concentration. Ref. = reference.

Purpose	Target	Primer Name	Primer Sequence (5′-3′)	Final Conc.	Size (bp)	Ref.
*Mycobacteria* speciation	*hsp*65	TB_*hsp*65-F	TGTAAAACGACGGCCAG TACCAACGATGGKGTKTCCAT	0.5 µM	401	[19]
		TB_*hsp*65-R	CAGGAAACAGCTATGAC CCTTGTCRAASCGCATRC	0.5 µM		
		XDR_NML_gyrA-2	GGGCTTCGGTGTACCTCAT	0.15 µM		
Macrolide resistance	*erm*(41)	ermF	GACCGGGGCCTTCTTCGTGAT	0.9 µM	632 (R) 356 (S)	[20]
		ermR1	GACTTCCCCGCACCGATTCC	0.9 µM		

**Table 3:** Contingency table (2 × 2) for *M. tuberculosis* complex (MTBC) samples that had a reference ID and passed *hsp*65 QC.

*M. tuberculosis* Complex		Reference ID	
		MTBC	Not MTBC
*hsp*65 ID	MTBC	47	0
	not MTBC	0	85

**Table 4:** Contingency table (2 × 2) for *M. abscesses* complex (MABC) samples that had a reference ID and passed *hsp*65 QC.

*M. abscesses* Complex		Reference ID	
		MABC	Not MABC
*hsp*65 ID	MABC	22	0
	not MABC	0	110

**Table 5:** Concordance by organism type for all validation samples that had a reference ID and passed *hsp*65 QC.

Organism	Total Samples	Sample Concordant	% Concordance
MTBC	47	47	100.0%
MABC	22	22	100.0%
NTM	46	44	95.7%
NM	17	17	100.0%
Total	132	129	98.5%

**Table 6:** Summary of LOD results for *M. tuberculosis* strain H37Rv (susceptible). Total replicates are indicated in brackets. Und = undetermined.

Dilution	Replicates Pass	MPT64 Ct
10^−2^	3 (3)	24.33
10^−3^	3 (3)	27.83
10^−4^	9 (9)	31.29
10^−5^	9 (9)	35.81
10^−6^	2 (3)	37.16
10^−7^	1 (3)	Und

**Table 7:** Summary of *hsp*65-based speciation LOD results in primary *M. tuberculosis* samples. Und = undetermined. NGS refers to samples flagged according to *hsp*65 speciation QC metrics.

Sample	MPT64 Ct	Smear Result	NGS	% ID	Size	Depth	*hsp*65 ID
PS1	31.32	2+	PASS	100.0	401	437	*Mycobacterium_tuberculosis*
PS2	Und	1 + (>10)	FAIL/REPEAT		0	0	
PS3	24.53	3+	PASS	100.0	401	445	*Mycobacterium_tuberculosis*
PS4	27.86	4+	PASS	99.8	401	422	*Mycobacterium_tuberculosis*
PS5	Und	1 + (<10)	FAIL/REPEAT		0	0	
PS6	39.26	1+	FAIL/REPEAT		0	0	
PS7	28.21	4+	PASS	100.0	401	440	*Mycobacterium_tuberculosis*
PS8	35.26	1 + (>10)	PASS	99.8	401	430	*Mycobacterium_tuberculosis*
PS9	27.29	4+	PASS	100.0	401	438	*Mycobacterium_tuberculosis*
PS10	37.32	1 + (<10)	FAIL/REPEAT	99.5	399	26	*Mycobacterium_tuberculosis*

**Table 8:** Failure rates for validation samples with an ID that did not pass *hsp*65 sequencing (i.e., NGS) and rescue rates for samples that failed Sanger sequencing (i.e., Sanger) but passed *hsp*65 sequencing (i.e., NGS). In both cases, passing refers to an assignment of a PASS flag based on QC metrics. NA = not applicable.

Organism	Samples with ID	Samples with ID Failing	Failure Rate	Samples Failing Sanger	Samples Passing NGS but Not Sanger	Rescue Rate
MTBC	53	6	11.3%	NA	NA	NA
MABC	22	0	0.0%	3	3	100.0%
NTM	47	2	4.3%	22	12	54.5%
NM	18	1	5.6%	21	12	57.1%
Unknown	NA	NA	NA	11	7	63.6%
Total	140	9	6.4%	57	34	59.6%

**Table 9:** Phylogenetic distribution of MAC members isolated in clinical samples over 18 months pre-NGS implementation and 18 months post-NGS implementation. Pre-NGS implementation, majority of MAC members were identified by MAC Hologic AccuProbe and small proportion by *hsp*65 Sanger sequencing; post-NGS implementation, all MAC members were identified by *hsp*65 NGS.

	Pre-NGS Implementation		Post-NGS Implementation	
	N	%	N	%
MAC	1165	93	0	0
*M. avium*	28	2.2	359	31
*M. intracellulare*	0	0	215	18.6
*M. paraintracellulare*	15	1.2	78	6.7
*M. chimaera*	16	1.3	116	10
*M. timonense*	28	2.2	363	31.4
*M. marseillense*	0	0	26	2.2
Total	1253	100	1157	100

**Table 10:** *erm*(41) resistance prediction based on erm amplicon size. MABC subspecies (subsp.) were identified through *hsp*65 speciation. The NGS column refers to *erm*(41) amplicon PASS metrics. Resistance refers to the call made by the pipeline, while Macrolide refers to the results of phenotypic susceptibility testing. Discrepancies are noted in red. Only samples that passed sequencing and had macrolide susceptibility results are shown here.

Sample	MABC Subsp.	NGS	Amplicon Size	Inducible Resistance Prediction	Phenotypic Macrolide Testing
Validation samples					
22A568	*abscessus*	PASS	632	resistant	resistant
22A616	*abscessus*	PASS	632	susceptible *	resistant
22A770	*abscessus*	PASS	632	resistant	resistant
22A879	*abscessus*	PASS	632	resistant	resistant
22A893	*abscessus*	PASS	632	resistant	resistant
22A809	*massiliense*	PASS	356	susceptible	susceptible
Clinical samples					
22H259	*abscessus*	PASS	632	susceptible *	susceptible
22H271	*abscessus*	PASS	632	resistant	resistant
22H282	*abscessus*	PASS	632	susceptible *	susceptible
22H292	*abscessus*	PASS	632	resistant	resistant
22H305	*abscessus*	PASS	632	resistant	resistant
22H450	*massiliense*	PASS	632	susceptible *	susceptible
22H476	*massiliense*	PASS	632	susceptible *	resistant
23H076	*abscessus*	PASS	632	resistant	resistant
23H462	*abscessus*	PASS	632	resistant	resistant
23H497	*abscessus*	PASS	632	resistant	resistant
23H642	*abscessus*	PASS	632	resistant	resistant
23H686	*abscessus*	PASS	632	resistant	resistant
23H727	*abscessus*	PASS	632	resistant	resistant
23H738	*abscessus*	PASS	632	resistant	resistant
23H742	*abscessus*	PASS	632	resistant	resistant
23H791	*abscessus*	PASS	632	resistant	resistant
23H811	*abscessus*	PASS	632	resistant	resistant
22H279	*massiliense*	PASS	356	susceptible	susceptible
22H289	*massiliense*	PASS	356	susceptible	susceptible
22H290	*massiliense*	PASS	356	susceptible	susceptible
22H303	*massiliense*	PASS	356	susceptible	susceptible
22H377	*massiliense*	PASS	356	susceptible	susceptible
22H383	*massiliense*	PASS	356	susceptible	susceptible
22H454	*massiliense*	PASS	356	susceptible	susceptible
22H492	*massiliense*	PASS	356	susceptible	susceptible
22H509	*massiliense*	PASS	356	susceptible	susceptible
22H544	*massiliense*	PASS	356	susceptible	susceptible
22H547	*massiliense*	PASS	356	susceptible	susceptible
22H632	*massiliense*	PASS	356	susceptible	susceptible
23H1167	*massiliense*	PASS	356	susceptible	susceptible
23H980	*massiliense*	PASS	356	susceptible	susceptible

* Isolates are determined to not have inducible macrolide resistance based on presence of T28C mutation in *erm*(41).

## Data Availability

Data is provided as part of this manuscript/Supplementary Materials; if any further information is required, please contact the corresponding author.

## Supplementary Materials

The following supporting information can be downloaded at https://www.mdpi.com/article/10.3390/tropicalmed10070192/s1, Figure S1: Alignment of *M. tuberculosis*, *M. caprae*, and *M. bovis*; Table S1: *Mycobacterium tuberculosis* complex samples used for validation. None of the samples were Sanger-sequenced; Table S2: *Mycobacterium abscessus* complex samples used for validation; Table S3: Non-tuberculous *Mycobacterium* (NTM) samples used for validation; Table S4: Non-mycobacterium samples used for validation; Table S5: Validation samples with no reference ID grouped by *HSPhsp*65 type. MABC = *Mycobacterium abscessus* complex; NTM = non-tuberculous *Mycobacterium*; NM = non-mycobacterium; Table S6: Additional information on direct patient samples used for validation. SPUT = sputum; BW = bronchial wash; TI = tissue; PLF = pleural fluid; BAL = bronchoalveolar lavage. IS6110 and MPT64 Ct values were only available for MTBC samples; Table S7: *Mycobacterium tuberculosis* complex samples sequenced post-implementation; Table S8: *Mycobacterium abscesses* complex samples sequenced post-implementation; Table S9: Non-tuberculous *Mycobacterium* samples sequenced post-implementation; Table S10: Non-mycobacterium samples sequenced post-implementation; Table S11: Post-implementation samples that could not be speciated by *hsp*65 due to poor sequencing; Table S12: Additional information for direct patient samples reviewed post-implementation. UND = undetermined. Neg = negative. BW = bronchial wash; CAP = proficiency sample designed to mimic sputum; SPUT = sputum; Scroll = scrolls cut from formalin-fixed paraffin-embedded tissue blocks (tissue source information not available); TI = tissue; TNP = test not performed; CSF = cerebrospinal fluid. * Culture set-up was completed in a reference lab; Table S13: H37Rv strain (susceptible) *hsp*65 speciation LOD and precision results. Table S14: erm amplicon and mutation information from M. abscesses validation samples. MABC subspecies (subsp.) was identified through *hsp*65 speciation. The NGS column refers to erm amplicon PASS metrics. Resistance refers to the call made by the pipeline, while Macrolide refers to testing of resistance. T28C mutation in bold; Table S15: erm amplicon and mutation information from M. abscesses clinical post-implementation samples. MABC subspecies (subsp.) was identified through *hsp*65 speciation. The NGS column refers to erm amplicon PASS metrics. Resistance refers to the call made by the pipeline, while Macrolide refers to testing of resistance. Discrepants are in red text. T28C mutation in bold.
