# Supplementary material for: Series 1: The Use of hsp65- and erm(41)-Targeted Amplicon Sequencing in the Diagnostic Workflow for Non-Tuberculous Mycobacteria
Source: Trop Med Infect Dis. 2025 Jul 9;10(7):192. doi: 10.3390/tropicalmed10070192 (PMC12298072; doi:10.3390/tropicalmed10070192)
Supplement: Supplementary file 1 [file tropicalmed-10-00192-s001.zip › tropicalmed-3645755-supplementary.pdf]

Supplementary Table S1. *Mycobacterium tuberculosis* complex samples used for validation. None of the samples were Sanger sequenced.

**Sample**	**NGS**	**% ID**	**Size**	**Depth**	**HSP65 ID**	**Reference ID**
Primary/direct samples						
PS1	PASS	100.0	401	437	*Mycobacterium_tuberculosis*	*Mycobacterium_tuberculosis*
PS3	PASS	100.0	401	445	*Mycobacterium_tuberculosis*	*Mycobacterium_tuberculosis*
PS4	PASS	99.8	401	422	*Mycobacterium_tuberculosis*	*Mycobacterium_tuberculosis*
PS7	PASS	100.0	401	440	*Mycobacterium_tuberculosis*	*Mycobacterium_tuberculosis*
PS8	PASS	99.8	401	430	*Mycobacterium_tuberculosis*	*Mycobacterium_tuberculosis*
PS9	PASS	100.0	401	438	*Mycobacterium_tuberculosis*	*Mycobacterium_tuberculosis*
SPEC-34	PASS	100.0	401	433	*Mycobacterium_tuberculosis*	*Mycobacterium_tuberculosis*
SPEC-35	PASS	100.0	401	391	*Mycobacterium_tuberculosis*	*Mycobacterium_tuberculosis*
SPEC-36	PASS	100.0	401	214	*Mycobacterium_tuberculosis*	*Mycobacterium_tuberculosis*
SPEC-37	PASS	99.8	401	434	*Mycobacterium_tuberculosis*	*Mycobacterium_tuberculosis*
SPEC-38	PASS	99.8	401	424	*Mycobacterium_tuberculosis*	*Mycobacterium_tuberculosis*
SPEC-39	PASS	100.0	401	421	*Mycobacterium_tuberculosis*	*Mycobacterium_tuberculosis*
SPEC-40	PASS	100.0	401	424	*Mycobacterium_tuberculosis*	*Mycobacterium_tuberculosis*
SPEC-41	PASS	100.0	401	384	*Mycobacterium_tuberculosis*	*Mycobacterium_tuberculosis*
SPEC-42	PASS	100.0	401	237	*Mycobacterium_tuberculosis*	*Mycobacterium_tuberculosis*
SPEC-44	PASS	100.0	401	215	*Mycobacterium_tuberculosis*	*Mycobacterium_tuberculosis*
PS10	FAIL/REPEAT	99.5	399	26	*Mycobacterium_tuberculosis*	*Mycobacterium_tuberculosis*
PS2	FAIL/REPEAT		0	0		*Mycobacterium_tuberculosis*
PS5	FAIL/REPEAT		0	0		*Mycobacterium_tuberculosis*
PS6	FAIL/REPEAT		0	0		*Mycobacterium_tuberculosis*
SPEC-43	FAIL/REPEAT	100.0	80	6	*Mycobacterium_tuberculosis*	*Mycobacterium_tuberculosis*
NML isolates						
NML-XDR-1	PASS	100.0	401	440	*Mycobacterium_tuberculosis*	*Mycobacterium_tuberculosis*
NML-XDR-10	PASS	100.0	401	427	*Mycobacterium_tuberculosis*	*Mycobacterium_tuberculosis*
NML-XDR-11	PASS	100.0	401	431	*Mycobacterium_tuberculosis*	*Mycobacterium_tuberculosis*
NML-XDR-12	PASS	100.0	401	428	*Mycobacterium_tuberculosis*	*Mycobacterium_tuberculosis*
NML-XDR-13	PASS	100.0	401	420	*Mycobacterium_tuberculosis*	*Mycobacterium_tuberculosis*
NML-XDR-14	PASS	100.0	401	424	*Mycobacterium_tuberculosis*	*Mycobacterium_tuberculosis*
NML-XDR-15	PASS	100.0	401	440	*Mycobacterium_tuberculosis*	*Mycobacterium_bovis_BCG*
NML-XDR-16	PASS	100.0	401	427	*Mycobacterium_tuberculosis*	*Mycobacterium_tuberculosis*
NML-XDR-17	PASS	100.0	401	445	*Mycobacterium_tuberculosis*	*Mycobacterium_tuberculosis*
NML-XDR-2	PASS	100.0	401	418	*Mycobacterium_tuberculosis*	*Mycobacterium_tuberculosis*

NML-XDR-4	PASS	99.8	401	440	*Mycobacterium_tuberculosis*	*Mycobacterium_tuberculosis*
NML-XDR-5	PASS	100.0	401	432	*Mycobacterium_tuberculosis*	*Mycobacterium_bovis_BCG*
NML-XDR-6	PASS	100.0	401	431	*Mycobacterium_tuberculosis*	*Mycobacterium_bovis_BCG*
NML-XDR-7	PASS	100.0	401	421	*Mycobacterium_tuberculosis*	*Mycobacterium_bovis_BCG*
NML-XDR-8	PASS	100.0	401	428	*Mycobacterium_tuberculosis*	*Mycobacterium_tuberculosis*
NML-XDR-9	PASS	99.8	401	437	*Mycobacterium_tuberculosis*	*Mycobacterium_tuberculosis*
NML-XDR-3	FAIL/REPEAT		0	0		MTBC
Culture samples						
18s262	PASS	100.0	401	428	*Mycobacterium_tuberculosis*	*Mycobacterium_tuberculosis*
18s423	PASS	99.8	401	431	*Mycobacterium_tuberculosis*	*Mycobacterium_tuberculosis*
18s514	PASS	99.8	401	420	*Mycobacterium_tuberculosis*	*Mycobacterium_tuberculosis*
19s078	PASS	100.0	401	440	*Mycobacterium_tuberculosis*	*Mycobacterium_tuberculosis*
20s291	PASS	100.0	401	426	*Mycobacterium_tuberculosis*	*Mycobacterium_tuberculosis*
20s304	PASS	100.0	401	427	*Mycobacterium_tuberculosis*	*Mycobacterium_tuberculosis*
22s250	PASS	100.0	401	425	*Mycobacterium_tuberculosis*	*Mycobacterium_tuberculosis*
22s291	PASS	100.0	401	425	*Mycobacterium_tuberculosis*	*Mycobacterium_tuberculosis*
22s305	PASS	99.8	401	430	*Mycobacterium_tuberculosis*	*Mycobacterium_tuberculosis*
22s317	PASS	100.0	401	436	*Mycobacterium_tuberculosis*	*Mycobacterium_tuberculosis*
22s320	PASS	100.0	401	436	*Mycobacterium_tuberculosis*	*Mycobacterium_tuberculosis*
22s431	PASS	100.0	401	437	*Mycobacterium_tuberculosis*	*Mycobacterium_tuberculosis*
23s071	PASS	99.8	401	438	*Mycobacterium_tuberculosis*	*Mycobacterium_tuberculosis*
23s134	PASS	99.8	401	439	*Mycobacterium_tuberculosis*	*Mycobacterium_tuberculosis*
23s137	PASS	99.8	401	443	*Mycobacterium_tuberculosis*	*Mycobacterium_tuberculosis*

Supplementary Table S2. *Mycobacterium abscessus* complex samples used for validation.

Sample	NGS	% ID	Size	Depth	HSP65 ID	Reference ID	Sanger
Culture samples							
SPEC-10	PASS	100	401	449	*Mycobacterium_abscessus*_subsp.*abscessus*	*Mycobacterium_abscessus*_subsp.*abscessus*	PASS
SPEC-101	PASS	100	401	458	*Mycobacterium_abscessus*_subsp.*abscessus*	*Mycobacterium_abscessus*_subsp.*abscessus*	PASS
SPEC-108	PASS	100	401	441	*Mycobacterium_abscessus*_subsp.*abscessus*	*Mycobacterium_abscessus*_subsp.*abscessus*	PASS
SPEC-12	PASS	100	401	440	*Mycobacterium_abscessus*_subsp.*abscessus*	*Mycobacterium_abscessus*_subsp.*abscessus*	PASS
SPEC-126	PASS	100	401	460	*Mycobacterium_abscessus*_subsp.*abscessus*	*Mycobacterium_abscessus*_subsp.*abscessus*	PASS
SPEC-145	PASS	100	401	463	*Mycobacterium_abscessus*_subsp.*massiliense*	*Mycobacterium_abscessus*_subsp.*massiliense*	PASS
SPEC-150	PASS	100	401	514	*Mycobacterium_abscessus*_subsp.*abscessus*	*Mycobacterium_abscessus*_subsp.*abscessus*	PASS
SPEC-165	PASS	100	401	501	*Mycobacterium_abscessus*_subsp.*abscessus*	*Mycobacterium_abscessus*_subsp.*abscessus*	PASS
SPEC-27	PASS	100	401	447	*Mycobacterium_abscessus*_subsp.*massiliense*	*Mycobacterium_abscessus*_subsp.*massiliense*	PASS

SPEC-89	PASS	100	401	456	*Mycobacterium_abscessus*_subsp.*massiliense*	*Mycobacterium_abscessus*_subsp.*massiliense*	PASS
SPEC-93	PASS	100	401	470	*Mycobacterium_abscessus*_subsp.*massiliense*	*Mycobacterium_abscessus*_subsp.*massiliense*	PASS
SPEC-94	PASS	100	401	467	*Mycobacterium_abscessus*_subsp.*abscessus*	*Mycobacterium_abscessus*_subsp.*abscessus*	PASS
SPEC-22	PASS	100	401	436	*Mycobacterium_abscessus*_subsp.*abscessus*	*Mycobacterium_abscessus*_subsp.*abscessus*	FAIL/REPEAT
SPEC-28	PASS	100	401	430	*Mycobacterium_abscessus*_subsp.*massiliense*	*Mycobacterium_abscessus*_subsp.*massiliense*	FAIL/REPEAT
SPEC-80	PASS	100	401	461	*Mycobacterium_abscessus*_subsp.*abscessus*	Forward sequence only - MASAB	FAIL/REPEAT
22A568	PASS	100	401	454	*Mycobacterium_abscessus*_subsp.*abscessus*	*M. abscessus* subspecies	
22A616	PASS	100	401	439	*Mycobacterium_abscessus*_subsp.*abscessus*	*M. abscessus* subspecies	
22A770	PASS	100	401	455	*Mycobacterium_abscessus*_subsp.*abscessus*	*M. abscessus* subspecies	
22A809	PASS	100	401	445	*Mycobacterium_abscessus*_subsp.*massiliense*	*M. abscessus* subspecies	
22A879	PASS	100	401	439	*Mycobacterium_abscessus*_subsp.*abscessus*	*M. abscessus* subspecies	
22A893	PASS	100	401	444	*Mycobacterium_abscessus*_subsp.*abscessus*	*M. abscessus* subspecies	
SPEC-60	PASS	100	401	432	*Mycobacterium_abscessus*_subsp.*abscessus*	*Mycobacterium_abscessus*_subsp.*abscessus*	
SPEC-61	PASS	100	401	443	*Mycobacterium_abscessus*_subsp.*bolletii*	*Mycobacterium_abscessus*_subsp.*bolletii*	
SPEC-62	PASS	100	401	422	*Mycobacterium_abscessus*_subsp.*massiliense*	*Mycobacterium_abscessus*_subsp.*massiliense*	
SPEC-63	PASS	100	401	440	*Mycobacterium_abscessus*_subsp.*abscessus*	*Mycobacterium_abscessus*_subsp.*abscessus*	

Supplementary Table S3. Non-tuberculous *Mycobacterium* (NTM) samples used for validation.

Sample	NGS	% ID	Size	Depth	HSP65 ID	Reference ID	Sanger
Primary/direct samples							
SPEC-45	PASS	100.0	401	436	*Mycobacterium_haemophilum*	*Mycobacterium_haemophilum*	
SPEC-47	PASS	100.0	401	435	*Mycobacterium_chelonae*	*Mycobacterium_chelonae*	
SPEC-48	PASS	100.0	401	241	*Mycobacterium_neoaurum*	*Mycobacterium_gordonae*	
SPEC-49	PASS	100.0	401	477	*Mycobacterium_brumae*	*Mycobacterium_brumae*	
SPEC-50	PASS	100.0	401	192	*Mycobacterium_avium*	*Mycobacterium_gordonae*	
SPEC-46	MIXED_BASES	99.5	401	121	*Mycobacterium_gordonae*_strain_CIP104529	*Mycobacterium_gordonae*	
SPEC-51	FAIL/REPEAT		0	0		*Mycobacterium_celatum*	
Culture samples							
SPEC-163	PASS	100.0	401	340	*Mycobacterium_fortuitum*_subsp.*fortuitum*	*Mycobacterium_fortuitum*_subsp.*fortuitum*	PASS
SPEC-14	PASS	99.5	401	414	*Mycobacterium_gordonae*_strain_CIP104529	*Mycobacterium_gordonae*_strain_CIP104529	PASS
SPEC-1	PASS	100.0	401	426	*Mycobacterium_poriferae*	*M. poriferae*_ATCC_35087T.seq	PASS
SPEC-100	PASS	100.0	401	432	*Mycobacterium_branderi*	*Mycobacterium_branderi*	PASS
SPEC-105	PASS	99.0	401	387	*Mycobacterium_phocaicum*	*Mycobacterium_phocaicum*	PASS
SPEC-106	PASS	100.0	401	396	*Mycobacterium_peregrinum*	*Mycobacterium_peregrinum*	PASS
SPEC-109	PASS	100.0	401	500	*Mycobacterium_gordonae*_strain_CIP104529	*Mycobacterium_gordonae*_strain_CIP104529	PASS
SPEC-111	PASS	98.3	401	504	*Mycobacterium_asiaticum*	*Mycobacterium_asiaticum*	PASS

SPEC-114	PASS	99.5	401	476	*Mycobacterium_gordonae*_strain_CIP104529	*Mycobacterium_gordonae*_strain_CIP104529	PASS
SPEC-115	PASS	99.3	401	484	*Mycobacterium_kumamotonense*	*Mycobacterium_kumamotonense*	PASS
SPEC-116	PASS	100.0	401	483	*Mycobacterium_intracellulare*	*Mycobacterium_paraintracellulare*	PASS
SPEC-117	PASS	100.0	401	433	*Mycobacterium_gordonae*_strain_CIP104529	*Mycobacterium_gordonae*_strain_CIP104529	PASS
SPEC-118	PASS	99.8	401	497	*Mycobacterium_chelonae*	*Mycobacterium_chelonae*	PASS
SPEC-122	PASS	100.0	401	445	*Mycobacterium_paragordonae*	*Mycobacterium_paragordonae*	PASS
SPEC-13	PASS	100.0	401	418	*Mycobacterium_haemophilum*	*Mycobacterium_haemophilum*	PASS
SPEC-133	PASS	100.0	401	346	*Mycobacterium_brisbanense*	*Mycobacterium_brisbanense*	PASS
SPEC-136	PASS	100.0	401	444	*Mycobacterium_paragordonae*	*Mycobacterium_paragordonae*	PASS
SPEC-147	PASS	99.8	401	354	*Mycobacterium_cosmeticum*	*Mycobacterium_cosmeticum*	PASS
SPEC-149	PASS	99.8	401	484	*Mycobacterium_chelonae*	*Mycobacterium_chelonae*	PASS
SPEC-152	PASS	100.0	401	480	*Mycobacterium_gordonae*_strain_CIP104529	*Mycobacterium_gordonae*_strain_CIP104529	PASS
SPEC-157	PASS	98.3	401	480	*Mycobacterium_asiaticum*	*Mycobacterium_asiaticum*	PASS
SPEC-158	PASS	100.0	401	506	*Mycobacterium_chelonae*	*Mycobacterium_chelonae*	PASS
SPEC-159	PASS	99.8	401	369	*Mycobacterium_septicum*	*Mycobacterium_septicum*	PASS
SPEC-169	PASS	100.0	401	359	*Mycobacterium_fortuitum*_subsp.*fortuitum*	*Mycobacterium_fortuitum*_subsp.*fortuitum*	PASS
SPEC-170	PASS	100.0	401	478	*Mycobacterium_paragordonae*	*Mycobacterium_paragordonae*	PASS
SPEC-171	PASS	100.0	401	352	*Mycobacterium_fortuitum*_subsp.*fortuitum*	*Mycobacterium_fortuitum*_subsp.*fortuitum*	PASS
SPEC-2	PASS	100.0	401	418	*Mycobacterium_avium*	*Mycobacterium_avium*	PASS
SPEC-23	PASS	100.0	401	419	*Mycobacterium_timonense*	*Mycobacterium_timonense*	PASS
SPEC-24	PASS	99.8	401	438	*Mycobacterium_chelonae*	*Mycobacterium_chelonae*	PASS
SPEC-26	PASS	100.0	401	426	*Mycobacterium_avium*	*Mycobacterium_avium*	PASS
SPEC-29	PASS	100.0	401	418	*Mycobacterium_gordonae_2*	*Mycobacterium_gordonae_2*	PASS
SPEC-3	PASS	100.0	401	420	*Mycobacterium_timonense*	*Mycobacterium_timonense*	PASS
SPEC-4	PASS	100.0	401	437	*Mycobacterium_timonense*	*Mycobacterium_timonense*	PASS
SPEC-6	PASS	99.8	401	422	*Mycobacterium_chelonae*	*Mycobacterium_chelonae*	PASS
SPEC-78	PASS	99.8	401	400	*Mycobacterium_brisbanense*	*Mycobacterium_brisbanense*	PASS
SPEC-85	PASS	100.0	401	431	*Mycobacterium_xenopi*	*Mycobacterium_xenopi*	PASS
SPEC-87	PASS	100.0	401	434	*Mycobacterium_chelonae*	*Mycobacterium_chelonae*	PASS
SPEC-88	PASS	100.0	401	461	*Mycobacterium_chelonae*	*Mycobacterium_chelonae*	PASS
SPEC-95	PASS	99.8	401	451	*Mycobacterium_chelonae*	*Mycobacterium_chelonae*	PASS
SPEC-98	PASS	99.0	401	349	*Mycobacterium_phocaicum*	*Mycobacterium_phocaicum*	PASS
SPEC-140	PASS	100.0	401	440	*Mycobacterium_paragordonae*	*Mycobacterium_paragordonae*	FAIL/REPEAT
SPEC-141	PASS	100.0	401	433	*Mycobacterium_gordonae*_strain_CIP104529	*Mycobacterium_gordonae*_strain_CIP104529	FAIL/REPEAT
SPEC-143	PASS	100.0	401	443	*Mycobacterium_paragordonae*	*Mycobacterium_paragordonae*	FAIL/REPEAT
SPEC-132	PASS	98.3	401	424	*Mycobacterium_asiaticum*	*Mycobacterium_asiaticum*	FAIL/REPEAT
SPEC-134	PASS	100.0	401	446	*Mycobacterium_chelonae*	*Mycobacterium_chelonae*	FAIL/REPEAT
SPEC-135	PASS	99.8	401	349	*Mycobacterium_septicum*	*Mycobacterium_septicum*	FAIL/REPEAT

SPEC-20	PASS	100.0	401	440	*Mycobacterium_chelonae*	*Mycobacterium_chelonae*	FAIL/REPEAT
SPEC-30	PASS	100.0	401	441	*Mycobacterium_kumamotonense*	*Mycobacterium_kumamotonense*	FAIL/REPEAT
SPEC-77	PASS	100.0	401	435	*Mycobacterium_timonense*	*Mycobacterium_timonense*	FAIL/REPEAT
SPEC-79	PASS	100.0	401	435	*Mycobacterium_branderi*	Forward sequence only -*M. branderi*	FAIL/REPEAT
SPEC-90	PASS	99.0	401	370	*Mycobacterium_phocaicum*	*Mycobacterium_phocaicum*	FAIL/REPEAT
SPEC-91	PASS	100.0	401	355	*Mycobacterium_peregrinum*	*Mycobacterium_peregrinum*	FAIL/REPEAT
SPEC-130	MIXED_BASES	98.8	401	475	*Mycobacterium_kumamotonense*	*Mycobacterium_pulveris*	FAIL/REPEAT
SPEC-102	REVIEW	95.8	401	461	*Mycobacterium_gordonae_2*	*Mycobacterium_gordonae_2*	FAIL/REPEAT
SPEC-154	REVIEW	95.8	401	457	*Mycobacterium_gordonae_2*	*Mycobacterium_gordonae_2*	FAIL/REPEAT
SPEC-168	REVIEW	96.8	401	461	*Mycobacterium_pulveris*	*Mycobacterium_pulveris*	FAIL/REPEAT
SPEC-107	FAIL/REPEAT	87.5	373	382	*Mycobacterium_timonense*	*Mycobacterium_alsense*	FAIL/REPEAT
SPEC-127	FAIL/REPEAT	87.0	369	416	*Mycobacterium_talmoniae*	*Mycobacterium_alsense*	FAIL/REPEAT
SPEC-146	FAIL/REPEAT	99.3	398	339	*Mycobacterium_fortuitum*_subsp.*fortuitum*	*Mycobacterium_fortuitum*_subsp.*fortuitum*	FAIL/REPEAT
SPEC-148	FAIL/REPEAT		0	2		*Mycobacterium_paraintracellulare*	FAIL/REPEAT
SPEC-15	FAIL/REPEAT	96.8	397	380	*Mycobacterium_paraense*	*Mycobacterium_alsense*	FAIL/REPEAT
SPEC-8	FAIL/REPEAT	97.1	216	6	*Mycobacterium_rutilum*	*Gordonia*_species	FAIL/REPEAT

Supplementary Table S4. Non-*mycobacterium* samples used for validation.

**Sample**	**NGS**	**% ID**	**Size**	**Depth**	**HSP65 ID**	**Reference ID**	**Sanger**
Culture samples							
SPEC-104	PASS	100.0	401	497	*Gordonia*_species_5	*Gordonia*_species_5	PASS
SPEC-11	PASS	100.0	401	417	*Tsukamurella_tyrosinosolvens*	*Tsukamurella_tyrosinosolvens*	PASS
SPEC-110	PASS	100.0	401	467	*Gordonia*_species_5	*Gordonia*_species_5	PASS
SPEC-112	PASS	98.3	401	488	*Gordonia*_species_5	*Gordonia*_species_5	PASS
SPEC-119	PASS	99.8	401	437	*Tsukamurella_tyrosinosolvens*	*Tsukamurella_tyrosinosolvens*	PASS
SPEC-120	PASS	99.8	401	447	*Tsukamurella_tyrosinosolvens*	*Tsukamurella_tyrosinosolvens*	PASS
SPEC-153	PASS	99.8	401	466	*Tsukamurella_tyrosinosolvens*	*Tsukamurella_tyrosinosolvens*	PASS
SPEC-155	PASS	99.8	401	467	*Tsukamurella_tyrosinosolvens*	*Tsukamurella_tyrosinosolvens*	PASS
SPEC-156	PASS	99.3	401	453	*Gordonia*_species_4	*Gordonia*_species_4	PASS
SPEC-16	PASS	99.5	401	413	*Tsukamurella_tyrosinosolvens*	*Tsukamurella_tyrosinosolvens*	PASS
SPEC-161	PASS	99.3	401	459	*Tsukamurella_tyrosinosolvens*	*Tsukamurella_tyrosinosolvens*	PASS
SPEC-166	PASS	99.3	401	459	*Tsukamurella_tyrosinosolvens*	*Tsukamurella_tyrosinosolvens*	PASS
SPEC-17	PASS	99.8	401	431	*Tsukamurella_tyrosinosolvens*	*Tsukamurella_tyrosinosolvens*	PASS
SPEC-173	PASS	98.3	401	479	*Gordonia*_species_5	*Gordonia*_species	PASS
SPEC-31	PASS	99.8	401	425	*Tsukamurella_tyrosinosolvens*	*Tsukamurella_tyrosinosolvens*	PASS
SPEC-7	PASS	99.5	401	426	*Tsukamurella_tyrosinosolvens*	*Tsukamurella_tyrosinosolvens*	PASS
SPEC-83	PASS	100.0	401	429	*Gordonia*_species_5	*Gordonia*_species_5	PASS

SPEC-84	MIXED_BASES	98.8	401	470	*Gordonia*_species_5	*Gordonia*_species_5	PASS
SPEC-113	PASS	98.8	401	466	*Gordonia*_species_4	*Nocardia_niwae*	FAIL/REPEAT
SPEC-124	PASS	99.8	401	441	*Tsukamurella_tyrosinosolvens*	no sequence	FAIL/REPEAT
SPEC-129	PASS	98.8	401	436	*Gordonia*_species_4	*Gordonia*_species_4	FAIL/REPEAT
SPEC-138	PASS	99.3	401	434	*Tsukamurella_tyrosinosolvens*	*Tsukamurella_tyrosinosolvens*	FAIL/REPEAT
SPEC-139	PASS	100.0	401	440	*Gordonia*_species_5	*Gordonia*_species_5	FAIL/REPEAT
SPEC-142	PASS	99.0	401	434	*Gordonia*_species_4	*Gordonia*_species_4	FAIL/REPEAT
SPEC-144	PASS	99.8	401	436	*Tsukamurella_tyrosinosolvens*	*Tsukamurella_tyrosinosolvens*	FAIL/REPEAT
SPEC-162	PASS	99.0	401	486	*Gordonia*_species_*4*	*Gordonia*_species_*4*	FAIL/REPEAT
SPEC-172	PASS	100.0	401	484	*Tsukamurella_inchonensis*	*Tsukamurella_inchonensis*	FAIL/REPEAT
SPEC-18	PASS	100.0	401	425	*Tsukamurella_pulmonis*	*Tsukamurella_pulmonis*	FAIL/REPEAT
SPEC-5	PASS	98.5	401	440	*Gordonia*_species_*2*	*Gordonia*_species_*2*	FAIL/REPEAT
SPEC-86	PASS	99.8	401	432	*Tsukamurella_tyrosinosolvens*	*Tsukamurella_tyrosinosolvens*	FAIL/REPEAT
SPEC-92	PASS	99.0	401	440	*Tsukamurella_tyrosinosolvens*	*Tsukamurella_tyrosinosolvens*	FAIL/REPEAT
SPEC-131	MIXED_BASES	99.3	401	428	*Tsukamurella_tyrosinosolvens*	*Tsukamurella_tyrosinosolvens*	FAIL/REPEAT
SPEC-103	FAIL/REPEAT		0	0		*Nocardia_wallacei*	FAIL/REPEAT
SPEC-128	FAIL/REPEAT	96.8	218	3	*Nocardia_seriolae*	*Gordonia*_species	FAIL/REPEAT
SPEC-167	FAIL/REPEAT	99.5	400	464	*Gordonia*_species	*Mycobacterium_cosmeticum*	FAIL/REPEAT
SPEC-82	FAIL/REPEAT		0	0		*Nocardia_wallacei*	FAIL/REPEAT
SPEC-9	FAIL/REPEAT	96.6	148	0	*Nocardia_yamanshiensis*	*Gordonia*_species	FAIL/REPEAT
SPEC-96	FAIL/REPEAT		0	0		*Rhodococcus*_species	FAIL/REPEAT
SPEC-97	FAIL/REPEAT	95.2	234	100	*Nocardia_seriolae*	*Gordonia*_species	FAIL/REPEAT
SPEC-99	FAIL/REPEAT	94.3	244	73	*Nocardia_seriolae*	*Gordonia*_species	FAIL/REPEAT

Supplementary Table S5. Validation samples with no reference ID grouped by HSP65 type. MABC = *Mycobacterium abscessus* complex, NTM = non-tuberculous *Mycobacterium*, NM = non-mycobacterium.

Sample	NGS	% ID	Size	Depth	Type	Source	HSP65 ID	Reference ID	Sanger
SPEC-125	PASS	100.0	401	464	MABC	Culture	*Mycobacterium_abscessus*_subsp.*abscessus*	No sequence	FAIL/REPEAT
SPEC-137	PASS	100.0	401	443	MABC	Culture	*Mycobacterium_abscessus*_subsp.*abscessus*	No sequence	FAIL/REPEAT
SPEC-160	PASS	100.0	401	502	MABC	Culture	*Mycobacterium_abscessus*_subsp.*abscessus*	No PCR product	FAIL/REPEAT
SPEC-21	PASS	100.0	401	452	MABC	Culture	*Mycobacterium_abscessus*_subsp.*abscessus*	No sequence	FAIL/REPEAT
SPEC-19	PASS	100.0	401	443	NTM	Culture	*Mycobacterium_chelonae*	Junk	FAIL/REPEAT
SPEC-123	REVIEW	95.8	401	445	NTM	Culture	*Mycobacterium_gordonae_2*	No Sequence	FAIL/REPEAT
SPEC-81	REVIEW	95.8	401	424	NTM	Culture	*Mycobacterium_gordonae_2*	No Sequence	FAIL/REPEAT

SPEC-121	PASS	99.8	401	445	NM	Culture	*Tsukamurella_tyrosinosolvens*	Did not assemble	FAIL/REPEAT
SPEC-124	PASS	99.8	401	441	NM	Culture	*Tsukamurella_tyrosinosolvens*	No sequence	FAIL/REPEAT
SPEC-32	REVIEW	95.8	401	250	NM	Culture	*Actinomadura*_species	Short Sequence	FAIL/REPEAT
SPEC-151	FAIL/REPEAT	99.8	400	463	NM	Culture	*Gordonia*_species	No sequence	FAIL/REPEAT

Supplementary Table S6. Additional information on direct patient samples used for validation. SPUT = sputum, BW = bronchial wash, TI = tissue, PLF = pleural fluid, BAL = bronchoalveolar lavage. IS6110 and MPT64 Ct values were only available for MTBC samples.

Sample	NGS	HSP65 ID	Smear	TB	MAC	Source	MPT64
Mycobacterium tuberculosis complex (MTBC)							
SPEC-34	PASS	*Mycobacterium_tuberculosis*	2+	POS	NEG	SPUT	31.63
SPEC-35	PASS	*Mycobacterium_tuberculosis*	1+(>10)	POS	NEG	BW	35.92
SPEC-36	PASS	*Mycobacterium_tuberculosis*	2+	POS	NEG	SPUT	30.65
SPEC-37	PASS	*Mycobacterium_tuberculosis*	2+	POS	NEG	TI	33.84
SPEC-38	PASS	*Mycobacterium_tuberculosis*	1+(>10)	POS	NEG	PLF	31.54
SPEC-39	PASS	*Mycobacterium_tuberculosis*	1+(>10)	POS	NEG	BW	32.67
SPEC-40	PASS	*Mycobacterium_tuberculosis*	2+	POS	NEG	SPUT	33.78
SPEC-41	PASS	*Mycobacterium_tuberculosis*	2+	POS	NEG	SPUT	34.77
SPEC-42	PASS	*Mycobacterium_tuberculosis*	2+	POS	NEG	SPUT	32.57
SPEC-44	PASS	*Mycobacterium_tuberculosis*	1+(>10)	POS	NEG	SPUT	35.10
SPEC-43	FAIL/REPEAT	*Mycobacterium_tuberculosis*	1+(<10)	POS	NEG	SPUT	34.38
Non-tuberculous *Mycobacterium* (NTM)							
SPEC-45	PASS	*Mycobacterium_haemophilum*	2+	NEG	NEG	TI	
SPEC-47	PASS	*Mycobacterium_chelonae*	2+	NEG	NEG	TI	
SPEC-48	PASS	*Mycobacterium_neoaurum*	1+(>10)	NEG	NEG	BAL	
SPEC-49	PASS	*Mycobacterium_brumae*	2+	NEG	NEG	SPUT	
SPEC-50	PASS	*Mycobacterium_avium*	1+(>10)	NEG	NEG	SPUT	
SPEC-53	PASS	*Mycobacterium_paraterrae*	2+	NEG	NEG	SPUT	
SPEC-46	MIXED_BASES	*Mycobacterium_gordonae_strain_CIP104529*	1+(>10)	NEG	NEG	SPUT	
SPEC-51	FAIL/REPEAT		1+(<10)	NEG	NEG	SPUT	

Supplementary Table S7. *Mycobacterium tuberculosis* complex samples sequenced post-implementation.

Sample	NGS	HSP65 ID	% ID	Size	Depth	Aligned reads	Type
CS_1	PASS	*Mycobacterium_tuberculosis*	99.8	401	415	1332	Direct
CS_2	PASS	*Mycobacterium_tuberculosis*	100.0	401	413	1354	Direct
CS_3	PASS	*Mycobacterium_tuberculosis*	99.8	401	423	1432	Direct
CS_4	PASS	*Mycobacterium_tuberculosis*	100.0	401	380	1028	Direct
CS_5	PASS	*Mycobacterium_tuberculosis*	100.0	401	373	1236	Direct
CS_6	PASS	*Mycobacterium_tuberculosis*	100.0	401	418	1310	Direct
CS_7	PASS	*Mycobacterium_tuberculosis*	100.0	401	390	1238	Direct
CS_8	PASS	*Mycobacterium_tuberculosis*	99.8	401	408	1332	Direct
CS_9	FAIL/REPEAT	*Mycobacterium_tuberculosis*	99.0	397	37	110	Direct
23H1076	PASS	*Mycobacterium_tuberculosis*	99.8	401	379	1218	Culture
23H1106	PASS	*Mycobacterium_tuberculosis*	100.0	401	416	1304	Culture
23H1107	PASS	*Mycobacterium_tuberculosis*	100.0	401	416	1330	Culture
23H1169	PASS	*Mycobacterium_tuberculosis*	99.8	401	420	1412	Culture
23H909	PASS	*Mycobacterium_tuberculosis*	100.0	401	232	690	Culture
23H911	PASS	*Mycobacterium_tuberculosis*	99.8	401	385	1230	Culture
23H915	PASS	*Mycobacterium_tuberculosis*	100.0	401	297	1074	Culture
23H974	PASS	*Mycobacterium_tuberculosis*	100.0	401	448	1434	Culture
24H61	PASS	*Mycobacterium_tuberculosis*	100.0	401	423	1414	Culture

Supplementary Table S8. *Mycobacterium abscesses* complex samples sequenced post-implementation.

Sample	NGS	HSP65 ID	% ID	Size	Depth	Aligned reads	Type
CS_10	PASS	*Mycobacterium_abscessus*_subsp.*abscessus*	100.0	401	139	436	Direct
CS_11	PASS	*Mycobacterium_abscessus*_subsp.*abscessus*	100.0	401	329	1024	Direct
22H259	PASS	*Mycobacterium_abscessus*_subsp.*abscessus*	100.0	401	414	1416	Culture
22H271	PASS	*Mycobacterium_abscessus*_subsp.*abscessus*	100.0	401	414	1430	Culture
22H279	PASS	*Mycobacterium_abscessus*_subsp.*massiliense*	100.0	401	419	1446	Culture
22H282	PASS	*Mycobacterium_abscessus*_subsp.*abscessus*	100.0	401	426	1426	Culture
22H290	PASS	*Mycobacterium_abscessus*_subsp.*massiliense*	100.0	401	428	1446	Culture
22H292	PASS	*Mycobacterium_abscessus*_subsp.*abscessus*	100.0	401	412	1440	Culture
22H303	PASS	*Mycobacterium_abscessus*_subsp.*massiliense*	99.8	401	469	1412	Culture
22H305	PASS	*Mycobacterium_abscessus*_subsp.*abscessus*	100.0	401	423	1444	Culture
22H377	PASS	*Mycobacterium_abscessus*_subsp.*massiliense*	100.0	401	429	1430	Culture

22H383	PASS	*Mycobacterium_abscessus*_subsp.*massiliense*	99.8	401	435	1420	Culture
22H450	PASS	*Mycobacterium_abscessus*_subsp.*massiliense*	99.5	401	422	1430	Culture
22H454	PASS	*Mycobacterium_abscessus*_subsp.*massiliense*	100.0	401	415	1440	Culture
22H476	PASS	*Mycobacterium_abscessus*_subsp.*massiliense*	99.5	401	422	1422	Culture
22H492	PASS	*Mycobacterium_abscessus*_subsp.*massiliense*	99.8	401	419	1424	Culture
22H509	PASS	*Mycobacterium_abscessus*_subsp.*massiliense*	100.0	401	426	1426	Culture
22H544	PASS	*Mycobacterium_abscessus*_subsp.*massiliense*	100.0	401	436	1440	Culture
22H547	PASS	*Mycobacterium_abscessus*_subsp.*massiliense*	100.0	401	427	1430	Culture
22H632	PASS	*Mycobacterium_abscessus*_subsp.*massiliense*	100.0	401	433	1430	Culture
23H076	PASS	*Mycobacterium_abscessus*_subsp.*abscessus*	99.8	401	452	1422	Culture
23H1005	PASS	*Mycobacterium_abscessus*_subsp.*massiliense*	100.0	401	424	1442	Culture
23H1030	PASS	*Mycobacterium_abscessus*_subsp.*abscessus*	100.0	401	463	1440	Culture
23H1053	PASS	*Mycobacterium_abscessus*_subsp.*massiliense*	100.0	401	411	1430	Culture
23H1054	PASS	*Mycobacterium_abscessus*_subsp.*abscessus*	100.0	401	419	1418	Culture
23H1069	PASS	*Mycobacterium_abscessus*_subsp.*massiliense*	99.8	401	434	1424	Culture
23H1070	PASS	*Mycobacterium_abscessus*_subsp.*abscessus*	100.0	401	459	1416	Culture
23H1104	PASS	*Mycobacterium_abscessus*_subsp.*abscessus*	100.0	401	413	1440	Culture
23H1139	PASS	*Mycobacterium_abscessus*_subsp.*abscessus*	100.0	401	424	1458	Culture
23H1147	PASS	*Mycobacterium_abscessus*_subsp.*abscessus*	100.0	401	434	1440	Culture
23H1167	PASS	*Mycobacterium_abscessus*_subsp.*massiliense*	100.0	401	428	1446	Culture
23H1168	PASS	*Mycobacterium_abscessus*_subsp.*abscessus*	100.0	401	429	1448	Culture
23H1172	PASS	*Mycobacterium_abscessus*_subsp.*abscessus*	100.0	401	473	1432	Culture
23H1205	PASS	*Mycobacterium_abscessus*_subsp.*abscessus*	100.0	401	431	1448	Culture
23H1229	PASS	*Mycobacterium_abscessus*_subsp.*massiliense*	100.0	401	431	1468	Culture
23H1240	PASS	*Mycobacterium_abscessus*_subsp.*abscessus*	100.0	401	426	1436	Culture
23H1243	PASS	*Mycobacterium_abscessus*_subsp.*abscessus*	100.0	401	412	1442	Culture
23H1244	PASS	*Mycobacterium_abscessus*_subsp.*abscessus*	100.0	401	433	1430	Culture
23H1247	PASS	*Mycobacterium_abscessus*_subsp.*massiliense*	100.0	401	425	1448	Culture
23H1259	PASS	*Mycobacterium_abscessus*_subsp.*massiliense*	100.0	401	449	1428	Culture
23H1260	PASS	*Mycobacterium_abscessus*_subsp.*massiliense*	100.0	401	438	1446	Culture
23H1277	PASS	*Mycobacterium_abscessus*_subsp.*abscessus*	100.0	401	418	1416	Culture
23H462	PASS	*Mycobacterium_abscessus*_subsp.*abscessus*	100.0	401	416	1426	Culture
23H497	PASS	*Mycobacterium_abscessus*_subsp.*abscessus*	100.0	401	427	1440	Culture
23H642	PASS	*Mycobacterium_abscessus*_subsp.*abscessus*	100.0	401	427	1434	Culture
23H727	PASS	*Mycobacterium_abscessus*_subsp.*abscessus*	100.0	401	427	1416	Culture
23H738	PASS	*Mycobacterium_abscessus*_subsp.*abscessus*	100.0	401	426	1426	Culture
23H742	PASS	*Mycobacterium_abscessus*_subsp.*abscessus*	100.0	401	427	1436	Culture
23H791	PASS	*Mycobacterium_abscessus*_subsp.*abscessus*	100.0	401	420	1424	Culture

23H811	PASS	*Mycobacterium_abscessus*_subsp.*abscessus*	100.0	401	422	1430	Culture
23H924	PASS	*Mycobacterium_abscessus*_subsp.*abscessus*	100.0	401	406	1400	Culture
23H927	PASS	*Mycobacterium_abscessus*_subsp.*massiliense*	99.8	401	416	1448	Culture
23H930	PASS	*Mycobacterium_abscessus*_subsp.*bolletii*	99.8	401	456	1402	Culture
23H943	PASS	*Mycobacterium_abscessus*_subsp.*abscessus*	100.0	401	468	1388	Culture
23H944	PASS	*Mycobacterium_abscessus*_subsp.*abscessus*	100.0	401	424	1404	Culture
23H979	PASS	*Mycobacterium_abscessus*_subsp.*abscessus*	100.0	401	452	1424	Culture
23H980	PASS	*Mycobacterium_abscessus*_subsp.*massiliense*	100.0	401	460	1434	Culture
23H997	PASS	*Mycobacterium_abscessus*_subsp.*massiliense*	99.8	401	421	1440	Culture
24H49	PASS	*Mycobacterium_abscessus*_subsp.*abscessus*	100.0	401	417	1434	Culture
24H56	PASS	*Mycobacterium_abscessus*_subsp.*massiliense*	100.0	401	427	1422	Culture
24H58	PASS	*Mycobacterium_abscessus*_subsp.*massiliense*	100.0	401	437	1474	Culture
24H67	PASS	*Mycobacterium_abscessus*_subsp.*abscessus*	100.0	401	433	1436	Culture
22H289	MIXED_BASES	*Mycobacterium_abscessus*_subsp.*massiliense*	99.8	401	521	1420	Culture
23H686	FAIL/REPEAT	*Mycobacterium_abscessus*_subsp.*abscessus*	100.0	396	471	1408	Culture
24H52	FAIL/REPEAT	*Mycobacterium_abscessus*_subsp.*abscessus*	100.0	396	497	1408	Culture

Supplementary Table S9. Non-tuberculous *Mycobacterium* samples sequenced post-implementation.

Sample	NGS	HSP65 ID	% ID	Size	Depth	Aligned reads	Type
CS_12	PASS	*Mycobacterium_chelonae*	100.0	401	433	1458	Direct
CS_13	PASS	*Mycobacterium_chelonae*	99.8	401	425	1432	Direct
CS_14	PASS	*Mycobacterium_avium*	100.0	401	72	244	Direct
23H1000	PASS	*Mycobacterium_intracellulare*	100.0	401	407	1478	Culture
23H1002	PASS	*Mycobacterium_avium*	100.0	401	405	1424	Culture
23H1003	PASS	*Mycobacterium_timonense*	100.0	401	392	1456	Culture
23H1006	PASS	*Mycobacterium_mageritense*	99.5	401	424	1414	Culture
23H1008	PASS	*Mycobacterium_fortuitum*_subsp.*fortuitum*	100.0	401	421	1388	Culture
23H1009	PASS	*Mycobacterium_avium*	100.0	401	435	1404	Culture
23H1010	PASS	*Mycobacterium_timonense*	100.0	401	398	1452	Culture
23H1012	PASS	*Mycobacterium_timonense*	100.0	401	412	1446	Culture
23H1013	PASS	*Mycobacterium_fortuitum*_subsp.*fortuitum*	100.0	401	417	1446	Culture
23H1014	PASS	*Mycobacterium_timonense*	100.0	401	413	1448	Culture
23H1018	PASS	*Mycobacterium_timonense*	100.0	401	413	1444	Culture
23H1020	PASS	*Mycobacterium_avium*	100.0	401	413	1432	Culture
23H1021	PASS	*Mycobacterium_timonense*	100.0	401	425	1450	Culture
23H1023	PASS	*Mycobacterium_avium*	100.0	401	404	1432	Culture

23H1024	PASS	*Mycobacterium_intracellulare*	100.0	401	414	1436	Culture
23H1025	PASS	*Mycobacterium_avium*	100.0	401	411	1434	Culture
23H1027	PASS	*Mycobacterium_septicum*	99.8	401	441	1436	Culture
23H1029	PASS	*Mycobacterium_timonense*	100.0	401	504	1422	Culture
23H1032	PASS	*Mycobacterium_chelonae*	100.0	401	452	1426	Culture
23H1033	PASS	*Mycobacterium_chimaera*	100.0	401	451	1440	Culture
23H1034	PASS	*Mycobacterium_avium*	100.0	401	442	1438	Culture
23H1035	PASS	*Mycobacterium_intracellulare*	100.0	401	442	1466	Culture
23H1036	PASS	*Mycobacterium_timonense*	100.0	401	442	1456	Culture
23H1037	PASS	*Mycobacterium_szulgai*	99.8	401	466	1424	Culture
23H1038	PASS	*Mycobacterium_timonense*	100.0	401	432	1458	Culture
23H1039	PASS	*Mycobacterium_chimaera*	100.0	401	421	1418	Culture
23H1042	PASS	*Mycobacterium_avium*	100.0	401	402	1438	Culture
23H1043	PASS	*Mycobacterium_timonense*	100.0	401	409	1420	Culture
23H1044	PASS	*Mycobacterium_intracellulare*	99.3	401	407	1464	Culture
23H1045	PASS	*Mycobacterium_chimaera*	100.0	401	410	1434	Culture
23H1047	PASS	*Mycobacterium_intracellulare*	100.0	401	411	1438	Culture
23H1048	PASS	*Mycobacterium_intracellulare*	100.0	401	412	1428	Culture
23H1049	PASS	*Mycobacterium_gordonae_strain*_CIP104529	100.0	401	410	1412	Culture
23H1050	PASS	*Mycobacterium_brisbanense*	98.8	401	416	1454	Culture
23H1051	PASS	*Mycobacterium_colombiense*	99.8	401	406	1434	Culture
23H1052	PASS	*Mycobacterium_paragordonae*	99.5	401	416	1450	Culture
23H1055	PASS	*Mycobacterium_intracellulare*	100.0	401	432	1442	Culture
23H1056	PASS	*Mycobacterium_intracellulare*	100.0	401	438	1440	Culture
23H1057	PASS	*Mycobacterium_timonense*	100.0	401	435	1450	Culture
23H1058	PASS	*Mycobacterium_obuense*	100.0	401	432	1468	Culture
23H1060	PASS	*Mycobacterium_conceptionense*	99.5	401	453	1428	Culture
23H1061	PASS	*Mycobacterium_paragordonae*	100.0	401	447	1406	Culture
23H1062	PASS	*Mycobacterium_timonense*	100.0	401	439	1464	Culture
23H1063	PASS	*Mycobacterium_timonense*	100.0	401	445	1430	Culture
23H1064	PASS	*Mycobacterium_chimaera*	100.0	401	445	1436	Culture
23H1066	PASS	*Mycobacterium_avium*	100.0	401	432	1440	Culture
23H1068	PASS	*Mycobacterium_intracellulare*	99.8	401	439	1462	Culture
23H1071	PASS	*Mycobacterium_timonense*	100.0	401	427	1384	Culture
23H1072	PASS	*Mycobacterium_asiaticum*	99.3	401	404	1432	Culture
23H1075	PASS	*Mycobacterium_xenopi*	100.0	401	396	1446	Culture
23H1077	PASS	*Mycobacterium_paragordonae*	100.0	401	398	1440	Culture
23H1078	PASS	*Mycobacterium_gordonae_2*	99.0	401	445	1414	Culture

23H1079	PASS	*Mycobacterium_timonense*	100.0	401	399	1448	Culture
23H1080	PASS	*Mycobacterium_paragordonae*	99.3	401	458	1396	Culture
23H1081	PASS	*Mycobacterium_paragordonae*	100.0	401	409	1452	Culture
23H1082	PASS	*Mycobacterium_avium*	100.0	401	398	1454	Culture
23H1084	PASS	*Mycobacterium_intracellulare*	100.0	401	410	1444	Culture
23H1086	PASS	*Mycobacterium_florentinum*	100.0	401	415	1448	Culture
23H1088	PASS	*Mycobacterium_avium*	100.0	401	407	1432	Culture
23H1089	PASS	*Mycobacterium_timonense*	100.0	401	398	1448	Culture
23H1090	PASS	*Mycobacterium_chelonae*	99.8	401	414	1442	Culture
23H1091	PASS	*Mycobacterium_gordonae_2*	100.0	401	409	1422	Culture
23H1092	PASS	*Mycobacterium_paragordonae*	99.3	401	419	1438	Culture
23H1093	PASS	*Mycobacterium_simiae*	99.0	401	401	1440	Culture
23H1094	PASS	*Mycobacterium_chimaera*	100.0	401	410	1448	Culture
23H1095	PASS	*Mycobacterium_intracellulare*	100.0	401	411	1470	Culture
23H1096	PASS	*Mycobacterium_chimaera*	100.0	401	411	1452	Culture
23H1097	PASS	*Mycobacterium_simiae*	99.3	401	407	1424	Culture
23H1098	PASS	*Mycobacterium_avium*	100.0	401	402	1436	Culture
23H1100	PASS	*Mycobacterium_avium*	100.0	401	394	1438	Culture
23H1101	PASS	*Mycobacterium_colombiense*	98.8	401	413	1448	Culture
23H1105	PASS	*Mycobacterium_fortuitum_*subsp.*fortuitum*	100.0	401	410	1448	Culture
23H1109	PASS	*Mycobacterium_avium*	100.0	401	393	1454	Culture
23H1110	PASS	*Mycobacterium_timonense*	100.0	401	403	1444	Culture
23H1112	PASS	*Mycobacterium_szulgai*	99.8	401	397	1442	Culture
23H1114	PASS	*Mycobacterium_avium*	100.0	401	413	1440	Culture
23H1117	PASS	*Mycobacterium_paragordonae*	100.0	401	418	1432	Culture
23H1118	PASS	*Mycobacterium_paragordonae*	100.0	401	415	1438	Culture
23H1121	PASS	*Mycobacterium_xenopi*	100.0	401	421	1454	Culture
23H1122	PASS	*Mycobacterium_paragordonae*	100.0	401	417	1424	Culture
23H1123	PASS	*Mycobacterium_timonense*	100.0	401	420	1440	Culture
23H1124	PASS	*Mycobacterium_gordonae_strain_*CIP104529	100.0	401	420	1438	Culture
23H1125	PASS	*Mycobacterium_gordonae_strain_*CIP104529	100.0	401	415	1424	Culture
23H1127	PASS	*Mycobacterium_timonense*	100.0	401	397	1406	Culture
23H1128	PASS	*Mycobacterium_avium*	100.0	401	403	1448	Culture
23H1129	PASS	*Mycobacterium_avium*	100.0	401	403	1454	Culture
23H1130	PASS	*Mycobacterium_chimaera*	100.0	401	422	1434	Culture
23H1131	PASS	*Mycobacterium_porcinum*	99.5	401	412	1454	Culture
23H1133	PASS	*Mycobacterium_mantenii*	100.0	401	413	1470	Culture
23H1137	PASS	*Mycobacterium_timonense*	100.0	401	392	1462	Culture

23H1138	PASS	*Mycobacterium_chimaera*	100.0	401	417	1442	Culture
23H1141	PASS	*Mycobacterium_paragordonae*	99.8	401	422	1464	Culture
23H1143	PASS	*Mycobacterium_timonense*	100.0	401	410	1456	Culture
23H1144	PASS	*Mycobacterium_intracellulare*	100.0	401	418	1442	Culture
23H1148	PASS	*Mycobacterium_avium*	100.0	401	400	1478	Culture
23H1149	PASS	*Mycobacterium_arupense*	100.0	401	434	1426	Culture
23H1151	PASS	*Mycobacterium_colombiense*	99.3	401	410	1434	Culture
23H1153	PASS	*Mycobacterium_fortuitum*_subsp.*fortuitum*	100.0	401	414	1460	Culture
23H1154	PASS	*Mycobacterium_paragordonae*	99.8	401	429	1452	Culture
23H1155	PASS	*Mycobacterium_timonense*	100.0	401	388	1448	Culture
23H1157	PASS	*Mycobacterium_avium*	100.0	401	406	1450	Culture
23H1159	PASS	*Mycobacterium_avium*	100.0	401	408	1442	Culture
23H1160	PASS	*Mycobacterium_avium*	100.0	401	404	1434	Culture
23H1162	PASS	*Mycobacterium_mucogenicum*	100.0	401	423	1426	Culture
23H1163	PASS	*Mycobacterium_timonense*	100.0	401	410	1450	Culture
23H1164	PASS	*Mycobacterium_avium*	100.0	401	399	1434	Culture
23H1165	PASS	*Mycobacterium_intracellulare*	100.0	401	416	1442	Culture
23H1171	PASS	*Mycobacterium_timonense*	100.0	401	449	1426	Culture
23H1173	PASS	*Mycobacterium_gordonae_strain*_CIP104529	99.5	401	452	1418	Culture
23H1174	PASS	*Mycobacterium_timonense*	100.0	401	459	1410	Culture
23H1175	PASS	*Mycobacterium_chimaera*	100.0	401	468	1426	Culture
23H1176	PASS	*Mycobacterium_chelonae*	99.8	401	468	1436	Culture
23H1177	PASS	*Mycobacterium_timonense*	100.0	401	454	1398	Culture
23H1178	PASS	*Mycobacterium_intracellulare*	99.8	401	452	1398	Culture
23H1180	PASS	*Mycobacterium_timonense*	100.0	401	461	1420	Culture
23H1181	PASS	*Mycobacterium_timonense*	100.0	401	451	1414	Culture
23H1182	PASS	*Mycobacterium_timonense*	100.0	401	458	1420	Culture
23H1185	PASS	*Mycobacterium_intracellulare*	100.0	401	463	1406	Culture
23H1187	PASS	*Mycobacterium_timonense*	100.0	401	416	1448	Culture
23H1188	PASS	*Mycobacterium_intracellulare*	100.0	401	435	1450	Culture
23H1189	PASS	*Mycobacterium_brisbanense*	100.0	401	418	1420	Culture
23H1190	PASS	*Mycobacterium_avium*	100.0	401	424	1452	Culture
23H1191	PASS	*Mycobacterium_paragordonae*	99.3	401	429	1442	Culture
23H1193	PASS	*Mycobacterium_fortuitum*_subsp.*fortuitum*	100.0	401	423	1460	Culture
23H1194	PASS	*Mycobacterium_intracellulare*	100.0	401	424	1446	Culture
23H1195	PASS	*Mycobacterium_avium*	100.0	401	413	1464	Culture
23H1197	PASS	*Mycobacterium_avium*	100.0	401	411	1472	Culture
23H1198	PASS	*Mycobacterium_fortuitum*_subsp.*fortuitum*	100.0	401	416	1432	Culture

23H1201	PASS	*Mycobacterium_chelonae*	99.8	401	421	1428	Culture
23H1206	PASS	*Mycobacterium_gordonae_strain*_CIP104529	99.8	401	407	1426	Culture
23H1207	PASS	*Mycobacterium_paraffinicum*	98.5	401	388	1432	Culture
23H1210	PASS	*Mycobacterium_avium*	100.0	401	397	1462	Culture
23H1213	PASS	*Mycobacterium_avium*	100.0	401	395	1462	Culture
23H1214	PASS	*Mycobacterium_intracellulare*	100.0	401	412	1454	Culture
23H1220	PASS	*Mycobacterium_timonense*	100.0	401	414	1456	Culture
23H1224	PASS	*Mycobacterium_timonense*	100.0	401	403	1488	Culture
23H1225	PASS	*Mycobacterium_avium*	100.0	401	413	1478	Culture
23H1226	PASS	*Mycobacterium_timonense*	100.0	401	415	1494	Culture
23H1227	PASS	*Mycobacterium_chimaera*	100.0	401	426	1450	Culture
23H1228	PASS	*Mycobacterium_intracellulare*	100.0	401	447	1444	Culture
23H1230	PASS	*Mycobacterium_intracellulare*	100.0	401	424	1466	Culture
23H1231	PASS	*Mycobacterium_timonense*	100.0	401	393	1478	Culture
23H1232	PASS	*Mycobacterium_avium*	100.0	401	417	1474	Culture
23H1233	PASS	*Mycobacterium_marseillense*	99.8	401	421	1456	Culture
23H1234	PASS	*Mycobacterium_chelonae*	99.8	401	440	1464	Culture
23H1237	PASS	*Mycobacterium_lentiflavum*	100.0	401	409	1460	Culture
23H1238	PASS	*Mycobacterium_timonense*	100.0	401	400	1480	Culture
23H1239	PASS	*Mycobacterium_timonense*	100.0	401	400	1484	Culture
23H1241	PASS	*Mycobacterium_timonense*	100.0	401	410	1452	Culture
23H1242	PASS	*Mycobacterium_intracellulare*	100.0	401	413	1466	Culture
23H1246	PASS	*Mycobacterium_septicum*	99.8	401	416	1430	Culture
23H1248	PASS	*Mycobacterium_timonense*	100.0	401	430	1446	Culture
23H1250	PASS	*Mycobacterium_celatum*	100.0	401	459	1436	Culture
23H1251	PASS	*Mycobacterium_timonense*	100.0	401	429	1442	Culture
23H1252	PASS	*Mycobacterium_colombiense*	99.8	401	431	1434	Culture
23H1253	PASS	*Mycobacterium_paragordonae*	99.3	401	439	1424	Culture
23H1254	PASS	*Mycobacterium_chelonae*	99.8	401	447	1434	Culture
23H1257	PASS	*Mycobacterium_heckeshornense*	100.0	401	461	1340	Culture
23H1258	PASS	*Mycobacterium_paragordonae*	100.0	401	441	1432	Culture
23H1262	PASS	*Mycobacterium_timonense*	100.0	401	418	1436	Culture
23H1263	PASS	*Mycobacterium_timonense*	100.0	401	412	1456	Culture
23H1265	PASS	*Mycobacterium_timonense*	100.0	401	411	1456	Culture
23H1266	PASS	*Mycobacterium_timonense*	100.0	401	388	1438	Culture
23H1267	PASS	*Mycobacterium_nebraskense*	99.8	401	405	1414	Culture
23H1270	PASS	*Mycobacterium_chelonae*	99.8	401	429	1442	Culture
23H1271	PASS	*Mycobacterium_gordonae_strain*_CIP104529	100.0	401	418	1412	Culture

23H1272	PASS	*Mycobacterium_gordonae_strain*_CIP104529	100.0	401	419	1424	Culture
23H1273	PASS	*Mycobacterium_chelonae*	100.0	401	427	1446	Culture
23H1274	PASS	*Mycobacterium_timonense*	100.0	401	413	1424	Culture
23H1275	PASS	*Mycobacterium_avium*	100.0	401	411	1426	Culture
23H1276	PASS	*Mycobacterium_intracellulare*	99.8	401	419	1432	Culture
23H1280	PASS	*Mycobacterium_timonense*	100.0	401	408	1442	Culture
23H1282	PASS	*Mycobacterium_fortuitum*_subsp.*fortuitum*	100.0	401	424	1430	Culture
23H906	PASS	*Mycobacterium_chelonae*	99.8	401	428	1416	Culture
23H907	PASS	*Mycobacterium_chelonae*	99.8	401	413	1452	Culture
23H908	PASS	*Mycobacterium_chelonae*	98.5	401	423	1426	Culture
23H910	PASS	*Mycobacterium_chimaera*	100.0	401	411	1452	Culture
23H913	PASS	*Mycobacterium_avium*	100.0	401	392	1428	Culture
23H916	PASS	*Mycobacterium_chelonae*	100.0	401	409	1434	Culture
23H917	PASS	*Mycobacterium_paragordonae*	99.5	401	387	1422	Culture
23H918	PASS	*Mycobacterium_peregrinum*	99.5	401	417	1454	Culture
23H919	PASS	*Mycobacterium_avium*	100.0	401	398	1448	Culture
23H920	PASS	*Mycobacterium_intracellulare*	100.0	401	402	1436	Culture
23H921	PASS	*Mycobacterium_timonense*	100.0	401	391	1436	Culture
23H922	PASS	*Mycobacterium_timonense*	100.0	401	396	1444	Culture
23H923	PASS	*Mycobacterium_timonense*	100.0	401	400	1416	Culture
23H925	PASS	*Mycobacterium_avium*	100.0	401	407	1432	Culture
23H926	PASS	*Mycobacterium_peregrinum*	100.0	401	413	1436	Culture
23H928	PASS	*Mycobacterium_avium*	100.0	401	389	1434	Culture
23H931	PASS	*Mycobacterium_intracellulare*	100.0	401	404	1446	Culture
23H932	PASS	*Mycobacterium_avium*	100.0	401	390	1432	Culture
23H933	PASS	*Mycobacterium_intracellulare*	99.8	401	415	1446	Culture
23H934	PASS	*Mycobacterium_avium*	100.0	401	402	1434	Culture
23H935	PASS	*Mycobacterium_intracellulare*	100.0	401	417	1410	Culture
23H936	PASS	*Mycobacterium_timonense*	100.0	401	396	1426	Culture
23H937	PASS	*Mycobacterium_chimaera*	100.0	401	412	1424	Culture
23H938	PASS	*Mycobacterium_timonense*	100.0	401	415	1428	Culture
23H939	PASS	*Mycobacterium_chelonae*	99.8	401	411	1438	Culture
23H940	PASS	*Mycobacterium_chimaera*	100.0	401	416	1466	Culture
23H941	PASS	*Mycobacterium_avium*	100.0	401	408	1446	Culture
23H942	PASS	*Mycobacterium_kansasii*	100.0	401	410	1432	Culture
23H947	PASS	*Mycobacterium_intracellulare*	100.0	401	443	1432	Culture
23H949	PASS	*Mycobacterium_scrofulaceum*	100.0	401	444	1410	Culture
23H950	PASS	*Mycobacterium_avium*	100.0	401	436	1432	Culture

23H951	PASS	*Mycobacterium_phocaicum*	99.0	401	439	1424	Culture
23H952	PASS	*Mycobacterium_porcinum*	99.5	401	437	1440	Culture
23H954	PASS	*Mycobacterium_timonense*	100.0	401	433	1424	Culture
23H955	PASS	*Mycobacterium_avium*	100.0	401	439	1434	Culture
23H957	PASS	*Mycobacterium_timonense*	100.0	401	435	1450	Culture
23H958	PASS	*Mycobacterium_avium*	100.0	401	434	1430	Culture
23H960	PASS	*Mycobacterium_chimaera*	100.0	401	440	1442	Culture
23H961	PASS	*Mycobacterium_timonense*	100.0	401	428	1430	Culture
23H962	PASS	*Mycobacterium_timonense*	100.0	401	441	1436	Culture
23H963	PASS	*Mycobacterium_paragordonae*	100.0	401	447	1424	Culture
23H964	PASS	*Mycobacterium_palustre*	99.8	401	431	1420	Culture
23H965	PASS	*Mycobacterium_avium*	100.0	401	424	1454	Culture
23H966	PASS	*Mycobacterium_avium*	100.0	401	429	1430	Culture
23H967	PASS	*Mycobacterium_chelonae*	99.8	401	451	1420	Culture
23H968	PASS	*Mycobacterium_aubagnense*	99.0	401	432	1426	Culture
23H969	PASS	*Mycobacterium_avium*	100.0	401	432	1446	Culture
23H970	PASS	*Mycobacterium_paragordonae*	100.0	401	454	1420	Culture
23H971	PASS	*Mycobacterium_timonense*	100.0	401	434	1430	Culture
23H972	PASS	*Mycobacterium_intracellulare*	100.0	401	430	1422	Culture
23H973	PASS	*Mycobacterium_avium*	100.0	401	446	1430	Culture
23H975	PASS	*Mycobacterium_timonense*	100.0	401	434	1444	Culture
23H976	PASS	*Mycobacterium_kansasii*	100.0	401	438	1440	Culture
23H977	PASS	*Mycobacterium_intracellulare*	100.0	401	441	1450	Culture
23H978	PASS	*Mycobacterium_timonense*	100.0	401	435	1438	Culture
23H981	PASS	*Mycobacterium_timonense*	100.0	401	411	1436	Culture
23H982	PASS	*Mycobacterium_intracellulare*	100.0	401	423	1434	Culture
23H983	PASS	*Mycobacterium_intracellulare*	99.3	401	418	1436	Culture
23H984	PASS	*Mycobacterium_lentiflavum*	100.0	401	419	1440	Culture
23H985	PASS	*Mycobacterium_intracellulare*	100.0	401	412	1428	Culture
23H986	PASS	*Mycobacterium_timonense*	100.0	401	414	1440	Culture
23H987	PASS	*Mycobacterium_timonense*	100.0	401	410	1428	Culture
23H988	PASS	*Mycobacterium_paragordonae*	100.0	401	412	1440	Culture
23H989	PASS	*Mycobacterium_intracellulare*	100.0	401	422	1468	Culture
23H990	PASS	*Mycobacterium_kumamotonense*	100.0	401	418	1416	Culture
23H992	PASS	*Mycobacterium_arupense*	100.0	401	434	1422	Culture
23H998	PASS	*Mycobacterium_chimaera*	100.0	401	414	1446	Culture
23H999	PASS	*Mycobacterium_xenopi*	100.0	401	414	1462	Culture
24H37	PASS	*Mycobacterium_gordonae_strain*_CIP104529	100.0	401	457	1362	Culture

24H38	PASS	*Mycobacterium_marseillense*	100.0	401	418	1438	Culture
24H39	PASS	*Mycobacterium_intracellulare*	100.0	401	428	1452	Culture
24H40	PASS	*Mycobacterium_intracellulare*	100.0	401	418	1440	Culture
24H41	PASS	*Mycobacterium_timonense*	100.0	401	414	1448	Culture
24H42	PASS	*Mycobacterium_gordonae_strain_*CIP104529	100.0	401	413	1446	Culture
24H43	PASS	*Mycobacterium_timonense*	100.0	401	410	1452	Culture
24H44	PASS	*Mycobacterium_avium*	100.0	401	418	1466	Culture
24H45	PASS	*Mycobacterium_avium*	100.0	401	400	1456	Culture
24H47	PASS	*Mycobacterium_gordonae_2*	100.0	401	420	1450	Culture
24H48	PASS	*Mycobacterium_timonense*	100.0	401	404	1442	Culture
24H50	PASS	*Mycobacterium_gordonae_strain_*CIP104529	99.5	401	412	1424	Culture
24H51	PASS	*Mycobacterium_intracellulare*	100.0	401	429	1388	Culture
24H53	PASS	*Mycobacterium_timonense*	100.0	401	409	1440	Culture
24H54	PASS	*Mycobacterium_avium*	100.0	401	410	1434	Culture
24H57	PASS	*Mycobacterium_intracellulare*	100.0	401	414	1454	Culture
24H59	PASS	*Mycobacterium_avium*	100.0	401	412	1454	Culture
24H60	PASS	*Mycobacterium_avium*	100.0	401	410	1450	Culture
24H62	PASS	*Mycobacterium_chelonae*	99.8	401	423	1442	Culture
24H63	PASS	*Mycobacterium_paraense*	97.5	401	412	1442	Culture
24H64	PASS	*Mycobacterium_timonense*	100.0	401	410	1446	Culture
24H65	PASS	*Mycobacterium_timonense*	100.0	401	405	1464	Culture
24H66	PASS	*Mycobacterium_avium*	100.0	401	413	1456	Culture
24H68	PASS	*Mycobacterium_intracellulare*	100.0	401	415	1432	Culture
24H69	PASS	*Mycobacterium_intracellulare*	99.3	401	404	1444	Culture
24H71	PASS	*Mycobacterium_timonense*	100.0	401	409	1454	Culture
23H1026	REVIEW	*Mycobacterium_madagascariense*	94.5	401	425	1446	Culture
23H1028	REVIEW	*Mycobacterium_gordonae_2*	95.8	401	428	1434	Culture
23H1074	REVIEW	*Mycobacterium_mantenii*	96.8	401	407	1432	Culture
23H1108	REVIEW	*Mycobacterium_gordonae_2*	94.0	401	458	1234	Culture
23H1132	REVIEW	*Mycobacterium_kumamotonense*	95.8	401	381	1434	Culture
23H1134	REVIEW	*Mycobacterium_gordonae_2*	94.0	401	445	1260	Culture
23H1140	REVIEW	*Mycobacterium_gordonae_2*	94.0	401	442	1208	Culture
23H1156	REVIEW	*Mycobacterium_paragordonae*	97.3	401	416	1458	Culture
23H1179	REVIEW	*Mycobacterium_moriokaense*	94.8	401	241	1120	Culture
23H1202	REVIEW	*Mycobacterium_kumamotonense*	95.8	401	401	1420	Culture
23H1204	REVIEW	*Mycobacterium_gordonae_2*	94.0	401	452	1224	Culture
23H1279	REVIEW	*Mycobacterium_moriokaense*	94.8	401	302	1260	Culture
23H991	REVIEW	*Mycobacterium_madagascariense*	94.5	401	402	1426	Culture

23H996	REVIEW	*Mycobacterium_gordonae_2*	95.8	401	351	1246	Culture
23H1001	MIXED_BASES	*Mycobacterium_timonense*	95.3	401	537	1394	Culture
23H1007	MIXED_BASES	*Mycobacterium_timonense*	99.8	401	398	1452	Culture
23H1019	MIXED_BASES	*Mycobacterium_chimaera*	97.3	401	453	1412	Culture
23H1031	MIXED_BASES	*Mycobacterium_chimaera*	97.5	401	479	1420	Culture
23H1126	MIXED_BASES	*Mycobacterium_intracellulare*	96.8	401	459	1434	Culture
23H1150	MIXED_BASES	*Mycobacterium_timonense*	95.0	401	478	1398	Culture
23H1158	MIXED_BASES	*Mycobacterium_intracellulare*	97.3	401	439	1450	Culture
23H1186	MIXED_BASES	*Mycobacterium_intracellulare*	99.8	401	456	1398	Culture
23H1281	MIXED_BASES	*Mycobacterium_avium*	98.8	401	417	1416	Culture
23H993	MIXED_BASES	*Mycobacterium_timonense*	99.8	401	412	1424	Culture
24H36	MIXED_BASES	*Mycobacterium_timonense*	99.0	401	419	1424	Culture
23H1059	FAIL/REPEAT	*Mycobacterium_rutilum*	97.1	216	5	350	Culture
23H1083	FAIL/REPEAT	*Mycobacterium_intracellulare*	98.5	396	424	1444	Culture
23H1087	FAIL/REPEAT	*Mycobacterium_chelonae*	99.5	395	471	1418	Culture
23H1111	FAIL/REPEAT	*Mycobacterium_avium*	98.5	399	419	1182	Culture
23H1135	FAIL/REPEAT	*Mycobacterium_intracellulare*	98.5	396	444	1434	Culture
23H1136	FAIL/REPEAT	*Mycobacterium_timonense*	99.2	397	469	1382	Culture
23H1217	FAIL/REPEAT	*Mycobacterium_rutilum*	97.6	216	0	214	Culture
23H1219	FAIL/REPEAT	*Mycobacterium_novocastrense*	97.7	171	0	222	Culture
23H1236	FAIL/REPEAT	*Mycobacterium_intracellulare*	98.0	393	29	86	Culture
23H1245	FAIL/REPEAT	*Mycobacterium_novocastrense*	97.6	170	0	290	Culture
23H956	FAIL/REPEAT	*Mycobacterium_intracellulare*	97.5	395	477	1412	Culture
23H959	FAIL/REPEAT	*Mycobacterium_chimaera*	99.3	400	505	1406	Culture
A1367	FAIL/REPEAT	*Mycobacterium_shinjukuense*	96.0	151	0	188	Culture

Supplementary Table S10. Non-mycobacterium samples sequenced post-implementation.

Sample	NGS	HSP65 ID	% ID	Size	Depth	Aligned reads	Type
23H100	PASS	*Tsukamurella_tyrosinosolvens*	99.0	401	414	1408	Culture
23H101	PASS	*Tsukamurella_pulmonis*	100.0	401	432	1404	Culture
23H101	PASS	*Tsukamurella_tyrosinosolvens*	99.8	401	420	1440	Culture
23H101	PASS	*Gordonia*_species_*4*	98.8	401	434	1422	Culture
23H1022	PASS	*Tsukamurella_tyrosinosolvens*	99.8	401	471	1336	Culture
23H1040	PASS	*Tsukamurella_tyrosinosolvens*	99.8	401	423	1432	Culture
23H1046	PASS	*Tsukamurella_tyrosinosolvens*	99.8	401	424	1406	Culture
23H1065	PASS	*Nocardia_sp.*OAHPP5092	100.0	401	445	1446	Culture

23H1067	PASS	*Tsukamurella_pulmonis*	100.0	401	441	1426	Culture
23H1099	PASS	*Gordonia*_species	100.0	401	415	1394	Culture
23H1102	PASS	*Tsukamurella_tyrosinosolvens*	99.8	401	423	1426	Culture
23H1103	PASS	*Tsukamurella_tyrosinosolvens*	99.8	401	416	1438	Culture
23H1113	PASS	*Gordonia*_species_*4*	99.3	401	409	1430	Culture
23H1142	PASS	*Tsukamurella_tyrosinosolvens*	99.8	401	429	1450	Culture
23H1145	PASS	*Nocardia_farcinica*	100.0	401	424	1456	Culture
23H1146	PASS	*Tsukamurella_tyrosinosolvens*	99.0	401	415	1454	Culture
23H1161	PASS	*Gordonia*_species_*2*	98.5	401	426	1386	Culture
23H1183	PASS	*Tsukamurella_tyrosinosolvens*	99.8	401	466	1408	Culture
23H1184	PASS	*Tsukamurella_tyrosinosolvens*	99.3	401	451	1412	Culture
23H1192	PASS	*Gordonia*_species_*5*	98.3	401	419	1432	Culture
23H1196	PASS	*Tsukamurella_tyrosinosolvens*	100.0	401	423	1444	Culture
23H1199	PASS	*Gordonia*_species_*4*	98.8	401	432	1452	Culture
23H1200	PASS	*Nocardia*_species_*flavorosea*	97.5	401	421	1470	Culture
23H1203	PASS	*Tsukamurella_tyrosinosolvens*	100.0	401	420	1424	Culture
23H1211	PASS	*Tsukamurella_tyrosinosolvens*	100.0	401	409	1438	Culture
23H1218	PASS	*Tsukamurella_tyrosinosolvens*	99.8	401	409	1438	Culture
23H1221	PASS	*Tsukamurella_tyrosinosolvens*	100.0	401	438	1446	Culture
23H1222	PASS	*Tsukamurella_tyrosinosolvens*	99.8	401	469	1340	Culture
23H1223	PASS	*Tsukamurella_tyrosinosolvens*	99.8	401	422	1444	Culture
23H1235	PASS	*Gordonia*_species_*4*	99.0	401	422	1476	Culture
23H1249	PASS	*Gordonia*_species_*3*	100.0	401	421	1436	Culture
23H1255	PASS	*Gordonia*_species_*5*	98.5	401	437	1454	Culture
23H1256	PASS	*Gordonia*_species	100.0	401	431	1456	Culture
23H1261	PASS	*Gordonia*_species_*2*	98.5	401	446	1474	Culture
23H1268	PASS	*Gordonia*_species_*3*	100.0	401	425	1438	Culture
23H1269	PASS	*Tsukamurella_paurometabola_2*	99.3	401	422	1418	Culture
23H1278	PASS	*Tsukamurella_tyrosinosolvens*	99.8	401	421	1424	Culture
23H912	PASS	*Tsukamurella_tyrosinosolvens*	99.3	401	431	1388	Culture
23H914	PASS	*Tsukamurella_tyrosinosolvens*	99.8	401	415	1430	Culture
23H929	PASS	*Tsukamurella_tyrosinosolvens*	99.8	401	420	1412	Culture
23H945	PASS	*Tsukamurella_paurometabola_2*	98.3	401	429	1272	Culture
23H946	PASS	*Tsukamurella_pulmonis*	100.0	401	418	1426	Culture
23H953	PASS	*Tsukamurella_tyrosinosolvens*	99.8	401	436	1432	Culture
23H995	PASS	*Gordonia*_species_*5*	100.0	401	415	1462	Culture
24H35	PASS	*Gordonia*_species_*2*	98.5	401	436	1478	Culture
24H46	PASS	*Gordonia*_species_*5*	98.3	401	416	1434	Culture

24H55	PASS	*Tsukamurella_tyrosinosolvens*	99.8	401	418	1426	Culture
24H70	PASS	*Tsukamurella_tyrosinosolvens*	99.8	401	433	1320	Culture
24H72	PASS	*Tsukamurella_tyrosinosolvens*	98.8	401	418	1440	Culture
23H1085	MIXED_BASES	*Tsukamurella_tyrosinosolvens*	99.3	401	413	1462	Culture
23H1215	MIXED_BASES	*Nocardia_farcinica*	98.0	401	422	1414	Culture
23H1041	FAIL/REPEAT	*Tsukamurella_tyrosinosolvens*	99.5	394	432	1396	Culture
23H1073	FAIL/REPEAT	*Nocardia_seriolae*	96.7	211	1	120	Culture
23H1115	FAIL/REPEAT	*Nocardia_seriolae*	96.7	211	2	218	Culture
23H1152	FAIL/REPEAT	*Nocardia_acidivorans*	95.9	148	0	82	Culture
23H1166	FAIL/REPEAT	*Nocardia_seriolae*	94.8	252	121	660	Culture
23H1264	FAIL/REPEAT	*Nocardia_seriolae*	94.7	245	145	676	Culture
23H948	FAIL/REPEAT	*Nocardia_acidivorans*	96.0	150	0	224	Culture

Supplementary Table S11. Post-implementation samples that could not be speciated by *hsp65* due to poor sequencing.

**Sample**	**NGS**	**HSP65 ID**	**% ID**	**Size**	**Depth**	**Aligned reads**	**Type**
CS_15	FAIL/REPEAT			0	0	0	Direct
CS_16	FAIL/REPEAT			0	0	0	Direct
CS_17	FAIL/REPEAT			0	0	0	Direct
CS_18	FAIL/REPEAT			0	0	0	Direct
CS_19	FAIL/REPEAT			0	0	0	Direct
CS_20	FAIL/REPEAT			0	3	12	Direct
CS_21	FAIL/REPEAT			0	0	0	Direct
CS_22	FAIL/REPEAT			0	0	0	Direct
22H497	FAIL/REPEAT			0	2	4	Culture
23H1017	FAIL/REPEAT			0	0	0	Culture
23H1116	FAIL/REPEAT			0	0	0	Culture
23H1119	FAIL/REPEAT			0	0	0	Culture
23H1120	FAIL/REPEAT			0	0	0	Culture
23H1170	FAIL/REPEAT			0	0	0	Culture
23H1208	FAIL/REPEAT			0	0	0	Culture
23H1209	FAIL/REPEAT			0	0	0	Culture
23H1212	FAIL/REPEAT			0	0	0	Culture
23H1216	FAIL/REPEAT			0	0	0	Culture
23H994	FAIL/REPEAT			0	0	0	Culture

Supplementary Table S12. Additional information for direct patient samples reviewed post-implementation. UND = undetermined. Neg = negative. BW = bronchial wash, CAP = proficiency sample designed to mimic sputum, SPUT = sputum, Scroll = scrolls cut from formalin-fixed paraffin-embedded tissue blocks (tissue source information not available), TI = tissue, TNP = test not performed, CSF = cerebrospinal fluid. *Culture set-up was completed in a reference lab.

Sample	NGS	HSP65 ID	Culture result	Smear	Source	MPT64 Ct
M. tuberculosis complex (MTBC)						
CS_1	PASS	*Mycobacterium_tuberculosis*	TB	negative	TI	TNP
CS_2	PASS	*Mycobacterium_tuberculosis*	TB	4+	SPUT	TNP
CS_3	PASS	*Mycobacterium_tuberculosis*	No culture setup	n/a	Scrolls	24.28
CS_4	PASS	*Mycobacterium_tuberculosis*	TB	1+	Gastric Aspirate	35.58
CS_5	PASS	*Mycobacterium_tuberculosis*	TB	4+	SPUT	TNP
CS_6	PASS	*Mycobacterium_tuberculosis*	TB	n/a	CAP	33.78
CS_7	PASS	*Mycobacterium_tuberculosis*	TB	n/a	CAP	34.61
CS_8	PASS	*Mycobacterium_tuberculosis*	TB	1+	BW	TNP
CS_9	FAIL/REPEAT	*Mycobacterium_tuberculosis*	TB	1+	SPUT	34.85
*M. abcessus subs*pecies *complex* (MABC)						
CS_10	PASS	*Mycobacterium_abscessus*_subsp.*abscessus*	*M.abscessus sub. abscessus*	4+	SPUT	Undetermined
CS_11	PASS	*Mycobacterium_abscessus*_subsp.*abscessus*	*M.abscessus sub. abscessus*	4+	SPUT	Undetermined
Non-tuberculous *Mycobacterium* (NTM)						
CS_12	PASS	*Mycobacterium_chelonae*	*M.chelonae*	4+	TI	Undetermined
CS_13	PASS	*Mycobacterium_chelonae*	*M.chelonae*	2+	TI	35.84
CS_14	PASS	*Mycobacterium_avium*	*M.avium*	Neg	TI	35.84
Negative/Unknown						
CS_15	FAIL/REPEAT		No culture setup	n/a	Scroll	Negative
CS_16	FAIL/REPEAT		No growth	1+	CSF	Negative
CS_17	FAIL/REPEAT		No culture setup	n/a	Scroll	TNP
CS_18	FAIL/REPEAT		No culture setup*	n/a	TI	TNP
CS_19	FAIL/REPEAT		No culture setup*	n/a	TI	TNP
CS_20	FAIL/REPEAT		No growth	3+	SPUT	TNP
CS_21	FAIL/REPEAT		No culture setup	N/A	Scroll	Negative
CS_22	FAIL/REPEAT		No culture set up	n/a	Scroll	Undetermined

Supplementary Table S13. H37Rv strain (susceptible) *hsp65* speciation LOD and precision results.

Sample	Dilution	Replicate	NGS	HSP65 ID	% ID	% Coverage	Size	Depth	Aligned reads
RUN 1									
H37Rv-2-a	2	a	PASS	*Mycobacterium_tuberculosis*	100	100	401	423	1372
H37Rv-2-b	2	b	PASS	*Mycobacterium_tuberculosis*	100	100	401	435	1308
H37Rv-2-c	2	c	PASS	*Mycobacterium_tuberculosis*	100	100	401	431	1372
H37Rv-3-a	3	a	PASS	*Mycobacterium_tuberculosis*	100	100	401	429	1402
H37Rv-3-b	3	b	PASS	*Mycobacterium_tuberculosis*	100	100	401	434	1384
H37Rv-3-c	3	c	PASS	*Mycobacterium_tuberculosis*	100	100	401	426	1366
H37Rv-4-a	4	a	PASS	*Mycobacterium_tuberculosis*	100	100	401	485	1400
H37Rv-4-b	4	b	PASS	*Mycobacterium_tuberculosis*	100	100	401	428	1298
H37Rv-4-c	4	c	PASS	*Mycobacterium_tuberculosis*	100	100	401	430	1338
H37Rv-5-a	5	a	PASS	*Mycobacterium_tuberculosis*	100	100	401	275	896
H37Rv-5-b	5	b	PASS	*Mycobacterium_tuberculosis*	100	100	401	428	1300
H37Rv-5-c	5	c	PASS	*Mycobacterium_tuberculosis*	100	100	401	424	1238
H37Rv-6-a	6	a	FAIL/REPEAT			0	0	0	0
H37Rv-6-b	6	b	PASS	*Mycobacterium_tuberculosis*	100	100	401	452	1376
H37Rv-6-c	6	c	PASS	*Mycobacterium_tuberculosis*	100	100	401	492	1450
H37Rv-7-a	7	a	PASS	*Mycobacterium_tuberculosis*	100	100	401	516	1492
H37Rv-7-b	7	b	FAIL/REPEAT			0	0	0	0
H37Rv-7-c	7	c	FAIL/REPEAT			0	0	0	0
RUN 2									
H37-4-a	4	a	PASS	*Mycobacterium_tuberculosis*	100	100	401	488	1456
H37-4-b	4	b	PASS	*Mycobacterium_tuberculosis*	100	100	401	467	1458
H37-4-c	4	c	PASS	*Mycobacterium_tuberculosis*	100	100	401	478	1416
H37-5-a	5	a	PASS	*Mycobacterium_tuberculosis*	100	100	401	411	1156
H37-5-b	5	b	PASS	*Mycobacterium_tuberculosis*	100	100	401	502	1438
H37-5-c	5	c	PASS	*Mycobacterium_tuberculosis*	100	100	401	499	1438
RUN 3									
P2-H37-4-a	4	a	PASS	*Mycobacterium_tuberculosis*	100	100	401	503	1442
P2-H37-4-b	4	b	PASS	*Mycobacterium_tuberculosis*	100	100	401	516	1460
P2-H37-4-c	4	c	PASS	*Mycobacterium_tuberculosis*	100	100	401	512	1446
P2-H37-5-a	5	a	PASS	*Mycobacterium_tuberculosis*	100	100	401	468	1372
P2-H37-5-b	5	b	PASS	*Mycobacterium_tuberculosis*	100	100	401	455	1366
P2-H37-5-c	5	c	PASS	*Mycobacterium_tuberculosis*	100	100	401	406	1146

Supplementary Table S14. erm amplicon and mutation information from *M. abscesses* validation samples. MABC subspecies (subsp.) was identified through *hsp65* speciation. NGS column refers to erm amplicon PASS metrics. Resistance refers to the call made by the pipeline while Macrolide refers to testing of resistance. T28C mutation in bold. Discrepants are in red text.

Sample	MABC subsp.	NGS	Amplicon Size	Resistance	Macrolide	Protein size	SNP profile
22A568	*abscessus*	PASS	632	resistant	resistant	173	C159T, G238A, A255G, C330A
22A616	*abscessus*	PASS	632	susceptible	resistant	173	**T28C**, A255G
22A770	*abscessus*	PASS	632	resistant	resistant	173	C159T, G168C, G238A, A255G, C330A
22A879	*abscessus*	PASS	632	resistant	resistant	173	C159T, G168C, G238A, A255G, C330A
22A893	*abscessus*	PASS	632	resistant	resistant	173	C159T, G238A, A255G, C330A
SPEC-10	*abscessus*	PASS	632	susceptible		173	**T28C**, A255G
SPEC-101	*abscessus*	PASS	632	resistant		173	C159T, G168C, G238A, A255G, C330A
SPEC-108	*abscessus*	PASS	632	susceptible		173	**T28C**, A255G
SPEC-12	*abscessus*	PASS	632	resistant		173	C159T, G168C, G238A, A255G, C330A
SPEC-125	*abscessus*	PASS	632	resistant		173	G279T, T336C
SPEC-126	*abscessus*	PASS	632	resistant		173	G279T, T336C
SPEC-137	*abscessus*	PASS	632	resistant		173	G279T, T336C
SPEC-150	*abscessus*	PASS	632	resistant		173	C159T, G238A, A255G, C330A
SPEC-160	*abscessus*	PASS	632	resistant		173	G279T, T336C
SPEC-165	*abscessus*	PASS	632	resistant		173	C159T, G238A, A255G, C330A
SPEC-21	*abscessus*	PASS	632	resistant		173	C159T, G168C, G238A, A255G, C330A
SPEC-22	*abscessus*	PASS	632	resistant		173	C159T, G168C, G238A, A255G, C330A
SPEC-60	*abscessus*	PASS	632	resistant		173	G279T, T336C
SPEC-61	*bolletii*	PASS	632	resistant		173	T231C, A255G, C258T, A312C, T336C, A414G
SPEC-63	*abscessus*	PASS	632	resistant		173	C159T, G168C, G238A, A255G, C258T, C330A
SPEC-80	*abscessus*	PASS	632	resistant		173	C159T, G168C, G238A, A255G, C330A
SPEC-94	*abscessus*	PASS	632	resistant		173	G279T, T336C
22A809	*massiliense*	PASS	356	susceptible	susceptible	81	C41A, A46G, GGC60G, G85T, C90T, G109A, A123G, CTGGCGCCAGGGTGCTAGCCGTCGAGCTGCATCC GGGGCGGGCTCGACACCTTCGTTCACGGTTTGCC GAGGAAGATGTCCGGGTAGCGGAAGCGGACCTA CTCGCCTTCCGGTGGCCGCGACGGCCATTTCGGG TGGTGGCGAGCCCGCCCTACCAAGTCACCAGCG CACTGATCCGGAGTCTCTTGACGCCGGAATCCCG GCTGCTGGCTGCCGACCTGGTGCTGCAGCGCGGG

							GCTGTGCACAAACATGCGAAGCGAGCACCTGTTCGCCAT155C, A438C, G466A
SPEC-145	*massiliense*	PASS	356	susceptible		81	C41A, A46G, GGC60G, G85T, C90T, G109A, A123G, CTGGCGCCAGGGTGCTAGCCGTCGAGCTGCATCCGGGGCGGGCTCGACACCTTCGTTCACGGTTTGCCGAGGAAGATGTCCGGGTAGCGGAAGCGGACCTACTCGCCTTCCGGTGGCCGCGACGGCCATTTCGGGTGGTGGCGAGCCCGCCCTACCAAGTCACCAGCGCACTGATCCGGAGTCTCTTGACGCCGGAATCCCGGCTGCTGGCTGCCGACCTGGTGCTGCAGCGCGGGGCTGTGCACAAACATGCGAAGCGAGCACCTGTTCGCCAT155C, A438C, G466A
SPEC-27	*massiliense*	PASS	356	susceptible		81	C41A, A46G, GGC60G, G85T, C90T, G109A, A123G, CTGGCGCCAGGGTGCTAGCCGTCGAGCTGCATCCGGGGCGGGCTCGACACCTTCGTTCACGGTTTGCCGAGGAAGATGTCCGGGTAGCGGAAGCGGACCTACTCGCCTTCCGGTGGCCGCGACGGCCATTTCGGGTGGTGGCGAGCCCGCCCTACCAAGTCACCAGCGCACTGATCCGGAGTCTCTTGACGCCGGAATCCCGGCTGCTGGCTGCCGACCTGGTGCTGCAGCGCGGGGCTGTGCACAAACATGCGAAGCGAGCACCTGTTCGCCAT155C, A438C
SPEC-28	*massiliense*	PASS	356	susceptible		81	C41A, A46G, GGC60G, G85T, C90T, G109A, A123G, CTGGCGCCAGGGTGCTAGCCGTCGAGCTGCATCCGGGGCGGGCTCGACACCTTCGTTCACGGTTTGCCGAGGAAGATGTCCGGGTAGCGGAAGCGGACCTACTCGCCTTCCGGTGGCCGCGACGGCCATTTCGGGTGGTGGCGAGCCCGCCCTACCAAGTCACCAGCGCACTGATCCGGAGTCTCTTGACGCCGGAATCCCGGCTGCTGGCTGCCGACCTGGTGCTGCAGCGCGGGGCTGTGCACAAACATGCGAAGCGAGCACCTGTTCGCCAT155C, A438C, G466A
SPEC-62	*massiliense*	PASS	356	susceptible		81	C41A, A46G, GGC60G, G85T, C90T, G109A, A123G, CTGGCGCCAGGGTGCTAGCCGTCGAGCTGCATCCGGGGCGGGCTCGACACCTTCGTTCACGGTTTGCCGAGGAAGATGTCCGGGTAGCGGAAGCGGACCTACTCGCCTTCCGGTGGCCGCGACGGCCATTTCGGGTGGTGGCGAGCCCGCCCTACCAAGTCACCAGCGCACTGATCCGGAGTCTCTTGACGCCGGAATCCCGGCTGCTGGCTGCCGACCTGGTGCTGCAGCGCGGG

		PASS					GCTGTGCACAAACATGCGAAGCGAGCACCTGTT CGCCAT155C, A438C, G466A
SPEC-89	*massiliense*	PASS	356	susceptible		81	C41A, A46G, GGC60G, G85T, C90T, G109A, A123G, CTGGCGCCAGGGTGCTAGCCGTCGAGCTGCATCC GGGGCGGGCTCGACACCTTCGTTCACGGTTTGCC GAGGAAGATGTCCGGGTAGCGGAAGCGGACCTA CTCGCCTTCCGGTGGCCGCGACGGCCATTTCGGG TGGTGGCGAGCCCGCCCTACCAAGTCACCAGCG CACTGATCCGGAGTCTCTTGACGCCGGAATCCCG GCTGCTGGCTGCCGACCTGGTGCTGCAGCGCGGG GCTGTGCACAAACATGCGAAGCGAGCACCTGTT CGCCAT155C, A438C
SPEC-93	*massiliense*	PASS	356	susceptible		81	C41A, A46G, GGC60G, G85T, C90T, G109A, A123G, CTGGCGCCAGGGTGCTAGCCGTCGAGCTGCATCC GGGGCGGGCTCGACACCTTCGTTCACGGTTTGCC GAGGAAGATGTCCGGGTAGCGGAAGCGGACCTA CTCGCCTTCCGGTGGCCGCGACGGCCATTTCGGG TGGTGGCGAGCCCGCCCTACCAAGTCACCAGCG CACTGATCCGGAGTCTCTTGACGCCGGAATCCCG GCTGCTGGCTGCCGACCTGGTGCTGCAGCGCGGG GCTGTGCACAAACATGCGAAGCGAGCACCTGTT CGCCAT155C, A438C, G466A

Supplementary Table S15. erm amplicon and mutation information from *M. abscesses* clinical post-implementation samples. MABC subspecies (subsp.) was identified through *hsp65* speciation. NGS column refers to erm amplicon PASS metrics. Resistance refers to the call made by the pipeline while Macrolide refers to testing of resistance. T28C mutation in bold.

Sample	MABC subsp.	NGS	Amplicon size	Resistance	Macrolide	Protein size	SNP profile
22H259	*abscessus*	PASS	632	susceptible	susceptible	173	**T28C**, A255G
22H271	*abscessus*	PASS	632	resistant	resistant	173	C159T, G168C, G238A, A255G, C330A
22H282	*abscessus*	PASS	632	susceptible	susceptible	173	**T28C**, A255G
22H292	*abscessus*	PASS	632	resistant	resistant	173	G279T, T336C
22H305	*abscessus*	PASS	632	resistant	resistant	173	A120G, G279T, T336C
22H450	*massiliense*	PASS	632	susceptible	susceptible	173	**T28C**, A255G

22H476	*massiliense*	PASS	632	susceptible	susceptible	173	**T28C**, A255G
23H076	*abscessus*	PASS	632	resistant	resistant	173	G279T, T336C
23H1030	*abscessus*	PASS	632	resistant		173	G279T, T336C
23H1054	*abscessus*	PASS	632	resistant		173	C159T, G168C, G238A, A255G, C330A
23H1070	*abscessus*	PASS	632	resistant		173	C159T, G238A, A255G, C330A
23H1104	*abscessus*	PASS	632	resistant		173	C159T, G238A, A255G, C330A
23H1139	*abscessus*	PASS	632	resistant		173	C159T, G168C, G238A, A255G, C330A
23H1147	*abscessus*	PASS	632	resistant		173	C159T, G168C, G238A, A255G, C330A
23H1168	*abscessus*	PASS	632	resistant		173	C159T, G168C, G238A, A255G, C330A
23H1172	*abscessus*	PASS	632	resistant		173	C159T, G168C, G238A, A255G, C330A
23H1205	*abscessus*	PASS	632	resistant		173	C159T, G168C, G238A, A255G, C330A
23H1240	*abscessus*	PASS	632	resistant		173	C159T, G168C, G238A, A255G, C330A
23H1243	*abscessus*	PASS	632	resistant		173	C159T, G168C, G238A, A255G, C330A
23H1244	*abscessus*	PASS	632	resistant		173	G238A, A255G, C419T
23H1277	*abscessus*	PASS	632	resistant		173	C159T, G238A, A255G, C330A
23H462	*abscessus*	PASS	632	resistant	resistant	173	C159T, G238A, A255G, C330A
23H497	*abscessus*	PASS	632	resistant	resistant	173	C159T, G238A, A255G, C330A
23H642	*abscessus*	PASS	632	resistant	resistant	173	C159T, G168C, G238A, A255G, C330A
23H686	*abscessus*	PASS	632	resistant	resistant	173	C159T, G238A, A255G, C330A
23H727	*abscessus*	PASS	632	resistant	resistant	173	C159T, G168C, G238A, A255G, C330A
23H738	*abscessus*	PASS	632	resistant	resistant	173	C159T, G238A, A255G, C330A
23H742	*abscessus*	PASS	632	resistant	resistant	173	C159T, G168C, G238A, A255G, C330A
23H791	*abscessus*	PASS	632	resistant	resistant	173	C159T, G238A, A255G, C330A
23H811	*abscessus*	PASS	632	resistant	resistant	173	C159T, G168C, G238A, A255G, C330A
23H924	*abscessus*	PASS	632	resistant		173	C159T, G238A, A255G, C330A
23H930	*bolletii*	PASS	632	susceptible		173	**T28C**, A255G
23H943	*abscessus*	PASS	632	resistant		173	G279T, T336C
23H944	*abscessus*	PASS	632	resistant		173	G238A, A255G, C419T
23H979	*abscessus*	PASS	632	resistant		173	G279T, T336C
24H49	*abscessus*	PASS	632	resistant		173	A120G, A255G
24H52	*abscessus*	PASS	632	resistant		173	C159T, G168C, G238A, A255G, C330A
24H67	*abscessus*	PASS	632	resistant		173	C159T, G238A, A255G, C330A
CS_10	*abscessus*	PASS	632	resistant		173	C159T, G168C, G238A, A255G, C330A
CS_11	*abscessus*	PASS	632	resistant		173	C159T, G168C, G238A, A255G, C330A
22H279	*massiliense*	PASS	356	susceptible	susceptible	81	C41A, A46G, GGC60G, G85T, C90T, G109A, A123G, CTGGCGCCAGGGTGCTAGCCGTCGAGCTGCATCCGGGGCGG GCTCGACACCTTCGTTCACGGTTTGCCGAGGAAGATGTCCG GGTAGCGGAAGCGGACCTACTCGCCTTCCGGTGGCCGCGAC GGCCATTTCGGGTGGTGGCGAGCCCGCCCTACCAAGTCACC

							AGCGCACTGATCCGGAGTCTCTTGACGCCGGAATCCCGGCT GCTGGCTGCCGACCTGGTGCTGCAGCGCGGGGCTGTGCACA AACATGCGAAGCGAGCACCTGTTCGCCAT155C, A438C, G466A
22H289	*massiliense*	PASS	356	susceptible	susceptible	81	C41A, A46G, GGC60G, G85T, C90T, G109A, A123G, CTGGCGCCAGGGTGCTAGCCGTCGAGCTGCATCCGGGGCGG GCTCGACACCTTCGTTCACGGTTTGCCGAGGAAGATGTCCG GGTAGCGGAAGCGGACCTACTCGCCTTCCGGTGGCCGCGAC GGCCATTTCGGGTGGTGGCGAGCCCGCCCTACCAAGTCACC AGCGCACTGATCCGGAGTCTCTTGACGCCGGAATCCCGGCT GCTGGCTGCCGACCTGGTGCTGCAGCGCGGGGCTGTGCACA AACATGCGAAGCGAGCACCTGTTCGCCAT155C, A438C, G466A
22H290	*massiliense*	PASS	356	susceptible	susceptible	81	C41A, A46G, GGC60G, G85T, C90T, G109A, A123G, CTGGCGCCAGGGTGCTAGCCGTCGAGCTGCATCCGGGGCGG GCTCGACACCTTCGTTCACGGTTTGCCGAGGAAGATGTCCG GGTAGCGGAAGCGGACCTACTCGCCTTCCGGTGGCCGCGAC GGCCATTTCGGGTGGTGGCGAGCCCGCCCTACCAAGTCACC AGCGCACTGATCCGGAGTCTCTTGACGCCGGAATCCCGGCT GCTGGCTGCCGACCTGGTGCTGCAGCGCGGGGCTGTGCACA AACATGCGAAGCGAGCACCTGTTCGCCAT155C, A438C, G466A
22H303	*massiliense*	PASS	356	susceptible	susceptible	81	C41A, A46G, GGC60G, G85T, C90T, G109A, A123G, CTGGCGCCAGGGTGCTAGCCGTCGAGCTGCATCCGGGGCGG GCTCGACACCTTCGTTCACGGTTTGCCGAGGAAGATGTCCG GGTAGCGGAAGCGGACCTACTCGCCTTCCGGTGGCCGCGAC GGCCATTTCGGGTGGTGGCGAGCCCGCCCTACCAAGTCACC AGCGCACTGATCCGGAGTCTCTTGACGCCGGAATCCCGGCT GCTGGCTGCCGACCTGGTGCTGCAGCGCGGGGCTGTGCACA AACATGCGAAGCGAGCACCTGTTCGCCAT155C, A438C
22H377	*massiliense*	PASS	356	susceptible	susceptible	81	C41A, A46G, GGC60G, G85T, C90T, G109A, A123G, CTGGCGCCAGGGTGCTAGCCGTCGAGCTGCATCCGGGGCGG GCTCGACACCTTCGTTCACGGTTTGCCGAGGAAGATGTCCG GGTAGCGGAAGCGGACCTACTCGCCTTCCGGTGGCCGCGAC GGCCATTTCGGGTGGTGGCGAGCCCGCCCTACCAAGTCACC AGCGCACTGATCCGGAGTCTCTTGACGCCGGAATCCCGGCT GCTGGCTGCCGACCTGGTGCTGCAGCGCGGGGCTGTGCACA AACATGCGAAGCGAGCACCTGTTCGCCAT155C, A438C, G466A

22H383	*massiliense*	PASS	356	susceptible	susceptible	81	C41A, A46G, GGC60G, G85T, C90T, G109A, A123G, CTGGCGCCAGGGTGCTAGCCGTCGAGCTGCATCCGGGGCGG GCTCGACACCTTCGTTCACGGTTTGCCGAGGAAGATGTCCG GGTAGCGGAAGCGGACCTACTCGCCTTCCGGTGGCCGCGAC GGCCATTTCGGGTGGTGGCGAGCCCGCCCTACCAAGTCACC AGCGCACTGATCCGGAGTCTCTTGACGCCGGAATCCCGGCT GCTGGCTGCCGACCTGGTGCTGCAGCGCGGGGCTGTGCACA AACATGCGAAGCGAGCACCTGTTCGCCAT155C, A438C
22H454	*massiliense*	PASS	356	susceptible	susceptible	81	C41A, A46G, GGC60G, G85T, C90T, G109A, A123G, CTGGCGCCAGGGTGCTAGCCGTCGAGCTGCATCCGGGGCGG GCTCGACACCTTCGTTCACGGTTTGCCGAGGAAGATGTCCG GGTAGCGGAAGCGGACCTACTCGCCTTCCGGTGGCCGCGAC GGCCATTTCGGGTGGTGGCGAGCCCGCCCTACCAAGTCACC AGCGCACTGATCCGGAGTCTCTTGACGCCGGAATCCCGGCT GCTGGCTGCCGACCTGGTGCTGCAGCGCGGGGCTGTGCACA AACATGCGAAGCGAGCACCTGTTCGCCAT155C, A438C, G466A
22H492	*massiliense*	PASS	356	susceptible	susceptible	81	C41A, A46G, GGC60G, G85T, C90T, G109A, A123G, CTGGCGCCAGGGTGCTAGCCGTCGAGCTGCATCCGGGGCGG GCTCGACACCTTCGTTCACGGTTTGCCGAGGAAGATGTCCG GGTAGCGGAAGCGGACCTACTCGCCTTCCGGTGGCCGCGAC GGCCATTTCGGGTGGTGGCGAGCCCGCCCTACCAAGTCACC AGCGCACTGATCCGGAGTCTCTTGACGCCGGAATCCCGGCT GCTGGCTGCCGACCTGGTGCTGCAGCGCGGGGCTGTGCACA AACATGCGAAGCGAGCACCTGTTCGCCAT155C,A438C
22H509	*massiliense*	PASS	356	susceptible	susceptible	81	C41A, A46G, GGC60G, G85T, C90T, G109A, A123G, CTGGCGCCAGGGTGCTAGCCGTCGAGCTGCATCCGGGGCGG GCTCGACACCTTCGTTCACGGTTTGCCGAGGAAGATGTCCG GGTAGCGGAAGCGGACCTACTCGCCTTCCGGTGGCCGCGAC GGCCATTTCGGGTGGTGGCGAGCCCGCCCTACCAAGTCACC AGCGCACTGATCCGGAGTCTCTTGACGCCGGAATCCCGGCT GCTGGCTGCCGACCTGGTGCTGCAGCGCGGGGCTGTGCACA AACATGCGAAGCGAGCACCTGTTCGCCAT155C, A438C, G466A
22H544	*massiliense*	PASS	356	susceptible	susceptible	81	C41A, A46G, GGC60G, G85T, C90T, G109A, A123G, CTGGCGCCAGGGTGCTAGCCGTCGAGCTGCATCCGGGGCGG GCTCGACACCTTCGTTCACGGTTTGCCGAGGAAGATGTCCG GGTAGCGGAAGCGGACCTACTCGCCTTCCGGTGGCCGCGAC GGCCATTTCGGGTGGTGGCGAGCCCGCCCTACCAAGTCACC AGCGCACTGATCCGGAGTCTCTTGACGCCGGAATCCCGGCT

							GCTGGCTGCCGACCTGGTGCTGCAGCGCGGGGCTGTGCACA AACATGCGAAGCGAGCACCTGTTCGCCAT155C, A438C, G466A
22H547	*massiliense*	PASS	356	susceptible	susceptible	81	C41A, A46G, GGC60G, G85T, C90T, G109A, A123G, CTGGCGCCAGGGTGCTAGCCGTCGAGCTGCATCCGGGGCGG GCTCGACACCTTCGTTCACGGTTTGCCGAGGAAGATGTCCG GGTAGCGGAAGCGGACCTACTCGCCTTCCGGTGGCCGCGAC GGCCATTTCGGGTGGTGGCGAGCCCGCCCTACCAAGTCACC AGCGCACTGATCCGGAGTCTCTTGACGCCGGAATCCCGGCT GCTGGCTGCCGACCTGGTGCTGCAGCGCGGGGCTGTGCACA AACATGCGAAGCGAGCACCTGTTCGCCAT155C, A438C, G466A
22H632	*massiliense*	PASS	356	susceptible	susceptible	81	C41A, A46G, GGC60G, G85T, C90T, G109A, A123G, CTGGCGCCAGGGTGCTAGCCGTCGAGCTGCATCCGGGGCGG GCTCGACACCTTCGTTCACGGTTTGCCGAGGAAGATGTCCG GGTAGCGGAAGCGGACCTACTCGCCTTCCGGTGGCCGCGAC GGCCATTTCGGGTGGTGGCGAGCCCGCCCTACCAAGTCACC AGCGCACTGATCCGGAGTCTCTTGACGCCGGAATCCCGGCT GCTGGCTGCCGACCTGGTGCTGCAGCGCGGGGCTGTGCACA AACATGCGAAGCGAGCACCTGTTCGCCAT155C, A438C, G466A
23H1005	*massiliense*	PASS	356	susceptible		81	C41A, A46G, GGC60G, G85T, C90T, G109A, A123G, CTGGCGCCAGGGTGCTAGCCGTCGAGCTGCATCCGGGGCGG GCTCGACACCTTCGTTCACGGTTTGCCGAGGAAGATGTCCG GGTAGCGGAAGCGGACCTACTCGCCTTCCGGTGGCCGCGAC GGCCATTTCGGGTGGTGGCGAGCCCGCCCTACCAAGTCACC AGCGCACTGATCCGGAGTCTCTTGACGCCGGAATCCCGGCT GCTGGCTGCCGACCTGGTGCTGCAGCGCGGGGCTGTGCACA AACATGCGAAGCGAGCACCTGTTCGCCAT155C, A438C, G466A
23H1053	*massiliense*	PASS	356	susceptible		81	C41A, A46G, GGC60G, G85T, C90T, G109A, A123G, CTGGCGCCAGGGTGCTAGCCGTCGAGCTGCATCCGGGGCGG GCTCGACACCTTCGTTCACGGTTTGCCGAGGAAGATGTCCG GGTAGCGGAAGCGGACCTACTCGCCTTCCGGTGGCCGCGAC GGCCATTTCGGGTGGTGGCGAGCCCGCCCTACCAAGTCACC AGCGCACTGATCCGGAGTCTCTTGACGCCGGAATCCCGGCT GCTGGCTGCCGACCTGGTGCTGCAGCGCGGGGCTGTGCACA AACATGCGAAGCGAGCACCTGTTCGCCAT155C, A438C, G466A

23H1069	*massiliense*	PASS	356	susceptible		81	C41A, A46G, GGC60G, G85T, C90T, G109A, A123G, CTGGCGCCAGGGTGCTAGCCGTCGAGCTGCATCCGGGGCGGGCTCGACACCTTCGTTCACGGTTTGCCGAGGAAGATGTCCGGGTAGCGGAAGCGGACCTACTCGCCTTCCGGTGGCCGCGACGGCCATTTCGGGTGGTGGCGAGCCCGCCCTACCAAGTCACCAGCGCACTGATCCGGAGTCTCTTGACGCCGGAATCCCGGCTGCTGGCTGCCGACCTGGTGCTGCAGCGCGGGGCTGTGCACAAACATGCGAAGCGAGCACCTGTTCGCCAT155C, A438C
23H1167	*massiliense*	PASS	356	susceptible	susceptible	81	C41A, A46G, GGC60G, G85T, C90T, G109A, A123G, CTGGCGCCAGGGTGCTAGCCGTCGAGCTGCATCCGGGGCGGGCTCGACACCTTCGTTCACGGTTTGCCGAGGAAGATGTCCGGGTAGCGGAAGCGGACCTACTCGCCTTCCGGTGGCCGCGACGGCCATTTCGGGTGGTGGCGAGCCCGCCCTACCAAGTCACCAGCGCACTGATCCGGAGTCTCTTGACGCCGGAATCCCGGCTGCTGGCTGCCGACCTGGTGCTGCAGCGCGGGGCTGTGCACAAACATGCGAAGCGAGCACCTGTTCGCCAT155C, A438C, G466A
23H1229	*massiliense*	PASS	356	susceptible		81	C41A, A46G, GGC60G, G85T, C90T, G109A, A123G,CTGGCGCCAGGGTGCTAGCCGTCGAGCTGCATCCGGGGCGGGCTCGACACCTTCGTTCACGGTTTGCCGAGGAAGATGTCCGGGTAGCGGAAGCGGACCTACTCGCCTTCCGGTGGCCGCGACGGCCATTTCGGGTGGTGGCGAGCCCGCCCTACCAAGTCACCAGCGCACTGATCCGGAGTCTCTTGACGCCGGAATCCCGGCTGCTGGCTGCCGACCTGGTGCTGCAGCGCGGGGCTGTGCACAAACATGCGAAGCGAGCACCTGTTCGCCAT155C, A438C, G466A
23H1247	*massiliense*	PASS	356	susceptible		81	C41A, A46G, GGC60G, G85T, C90T, G109A, A123G, CTGGCGCCAGGGTGCTAGCCGTCGAGCTGCATCCGGGGCGGGCTCGACACCTTCGTTCACGGTTTGCCGAGGAAGATGTCCGGGTAGCGGAAGCGGACCTACTCGCCTTCCGGTGGCCGCGACGGCCATTTCGGGTGGTGGCGAGCCCGCCCTACCAAGTCACCAGCGCACTGATCCGGAGTCTCTTGACGCCGGAATCCCGGCTGCTGGCTGCCGACCTGGTGCTGCAGCGCGGGGCTGTGCACAAACATGCGAAGCGAGCACCTGTTCGCCAT155C, A438C, G466A
23H1259	*massiliense*	PASS	356	susceptible		81	C41A, A46G, GGC60G, G85T, C90T, G109A, A123G, CTGGCGCCAGGGTGCTAGCCGTCGAGCTGCATCCGGGGCGGGCTCGACACCTTCGTTCACGGTTTGCCGAGGAAGATGTCCGGGTAGCGGAAGCGGACCTACTCGCCTTCCGGTGGCCGCGACGGCCATTTCGGGTGGTGGCGAGCCCGCCCTACCAAGTCACC

		PASS					AGCGCACTGATCCGGAGTCTCTTGACGCCGGAATCCCGGCT GCTGGCTGCCGACCTGGTGCTGCAGCGCGGGGCTGTGCACA AACATGCGAAGCGAGCACCTGTTCGCCAT155C, A438C, G466A
23H1260	*massiliense*	PASS	356	susceptible		81	C41A, A46G, GGC60G, G85T, C90T, G109A, A123G, CTGGCGCCAGGGTGCTAGCCGTCGAGCTGCATCCGGGGCGG GCTCGACACCTTCGTTCACGGTTTGCCGAGGAAGATGTCCG GGTAGCGGAAGCGGACCTACTCGCCTTCCGGTGGCCGCGAC GGCCATTTCGGGTGGTGGCGAGCCCGCCCTACCAAGTCACC AGCGCACTGATCCGGAGTCTCTTGACGCCGGAATCCCGGCT GCTGGCTGCCGACCTGGTGCTGCAGCGCGGGGCTGTGCACA AACATGCGAAGCGAGCACCTGTTCGCCAT155C, A438C, G466A
23H927	*massiliense*	PASS	356	susceptible		81	C41A, A46G, GGC60G, G85T, C90T, G109A, A123G, CTGGCGCCAGGGTGCTAGCCGTCGAGCTGCATCCGGGGCGG GCTCGACACCTTCGTTCACGGTTTGCCGAGGAAGATGTCCG GGTAGCGGAAGCGGACCTACTCGCCTTCCGGTGGCCGCGAC GGCCATTTCGGGTGGTGGCGAGCCCGCCCTACCAAGTCACC AGCGCACTGATCCGGAGTCTCTTGACGCCGGAATCCCGGCT GCTGGCTGCCGACCTGGTGCTGCAGCGCGGGGCTGTGCACA AACATGCGAAGCGAGCACCTGTTCGCCAT155C, A438C
23H980	*massiliense*	PASS	356	susceptible	susceptible	81	C41A, A46G, GGC60G, G85T, C90T, G109A, A123G, CTGGCGCCAGGGTGCTAGCCGTCGAGCTGCATCCGGGGCGG GCTCGACACCTTCGTTCACGGTTTGCCGAGGAAGATGTCCG GGTAGCGGAAGCGGACCTACTCGCCTTCCGGTGGCCGCGAC GGCCATTTCGGGTGGTGGCGAGCCCGCCCTACCAAGTCACC AGCGCACTGATCCGGAGTCTCTTGACGCCGGAATCCCGGCT GCTGGCTGCCGACCTGGTGCTGCAGCGCGGGGCTGTGCACA AACATGCGAAGCGAGCACCTGTTCGCCAT155C, A438C
23H997	*massiliense*	PASS	356	susceptible		81	C41A, A46G, GGC60G, G85T, C90T, G109A, A123G, CTGGCGCCAGGGTGCTAGCCGTCGAGCTGCATCCGGGGCGG GCTCGACACCTTCGTTCACGGTTTGCCGAGGAAGATGTCCG GGTAGCGGAAGCGGACCTACTCGCCTTCCGGTGGCCGCGAC GGCCATTTCGGGTGGTGGCGAGCCCGCCCTACCAAGTCACC AGCGCACTGATCCGGAGTCTCTTGACGCCGGAATCCCGGCT GCTGGCTGCCGACCTGGTGCTGCAGCGCGGGGCTGTGCACA AACATGCGAAGCGAGCACCTGTTCGCCAT155C, A438C, G466A, C497T
24H56	*massiliense*	PASS	356	susceptible		81	C41A, A46G, GGC60G, G85T, C90T, G109A, A123G, CTGGCGCCAGGGTGCTAGCCGTCGAGCTGCATCCGGGGCGG

							GCTCGACACCTTCGTTCACGGTTTGCCGAGGAAGATGTCCG GGTAGCGGAAGCGGACCTACTCGCCTTCCGGTGGCCGCGAC GGCCATTTCGGGTGGTGGCGAGCCCGCCCTACCAAGTCACC AGCGCACTGATCCGGAGTCTCTTGACGCCGGAATCCCGGCT GCTGGCTGCCGACCTGGTGCTGCAGCGCGGGGCTGTGCACA AACATGCGAAGCGAGCACCTGTTCGCCAT155C, A438C, G466A
24H58	*massiliense*	PASS	356	susceptible		81	C41A, A46G, GGC60G, G85T, C90T, G109A, A123G, CTGGCGCCAGGGTGCTAGCCGTCGAGCTGCATCCGGGGCGG GCTCGACACCTTCGTTCACGGTTTGCCGAGGAAGATGTCCG GGTAGCGGAAGCGGACCTACTCGCCTTCCGGTGGCCGCGAC GGCCATTTCGGGTGGTGGCGAGCCCGCCCTACCAAGTCACC AGCGCACTGATCCGGAGTCTCTTGACGCCGGAATCCCGGCT GCTGGCTGCCGACCTGGTGCTGCAGCGCGGGGCTGTGCACA AACATGCGAAGCGAGCACCTGTTCGCCAT155C, A438C, G466A

```
                  1         10        20        30        40        50        60
                  |         |         |         |         |         |         |
       M.bovis  CGCCAAGGAGATCGAGCTGGAGGATCCGTACGAGAAGATCGGCGCCGAGCTGGTCAAAGA
      M.caprae  CGCCAAGGAGATCGAGCTGGAGGATCCGTACGAGAAGATCGGCGCCGAGCTGGTCAAAGA
M.tuberculosis  CGCCAAGGAGATCGAGCTGGAGGATCCGTACGAGAAGATCGGCGCCGAGCTGGTCAAAGA

       M.bovis  GGTAGCCAAGAAGACCGATGACGTCGCCGGTGACGGCACCACGACGGCCACCGTGCTGGC
      M.caprae  GGTAGCCAAGAAGACCGATGACGTCGCCGGTGACGGCACCACGACGGCCACCGTGCTGGC
M.tuberculosis  GGTAGCCAAGAAGACCGATGACGTCGCCGGTGACGGCACCACGACGGCCACCGTGCTGGC

       M.bovis  CCAGGCGTTGGTTCGCGAGGGCCTGCGCAACGTCGCGGCCGGCGCCAACCCGCTCGGTCT
      M.caprae  CCAGGCGTTGGTTCGCGAGGGCCTGCGCAACGTCGCGGCCGGCGCCAACCCGCTCGGTCT
M.tuberculosis  CCAGGCGTTGGTTCGCGAGGGCCTGCGCAACGTCGCGGCCGGCGCCAACCCGCTCGGTCT

       M.bovis  CAAACGCGGCATCGAAAAGGCCGTGGAGAAGGTCACCGAGACCCTGCTCAAGGGCGCCAA
      M.caprae  CAAACGCGGCATCGAAAAGGCCGTGGAGAAGGTCACCGAGACCCTGCTCAAGGGCGCCAA
M.tuberculosis  CAAACGCGGCATCGAAAAGGCCGTGGAGAAGGTCACCGAGACCCTGCTCAAGGGCGCCAA

       M.bovis  GGAGGTCGAGACCAAGGAGCAGATTGCGGCCACCGCAGCGATTTCGGCGGGTGACCAGTC
      M.caprae  GGAGGTCGAGACCAAGGAGCAGATTGCGGCCACCGCAGCGATTTCGGCGGGTGACCAGTC
M.tuberculosis  GGAGGTCGAGACCAAGGAGCAGATTGCGGCCACCGCAGCGATTTCGGCGGGTGACCAGTC

       M.bovis  CATCGGTGACCTGATCGCCGAGGCGATGGACAAGGTGGGCAACGAGGGCGTCATCACCGT
      M.caprae  CATCGGTGACCTGATCGCCGAGGCGATGGACAAGGTGGGCAACGAGGGCGTCATCACCGT
M.tuberculosis  CATCGGTGACCTGATCGCCGAGGCGATGGACAAGGTGGGCAACGAGGGCGTCATCACCGT

       M.bovis  CGAGGAGTCCAACACCTTTGGGCTGCAGCTCGAGCTCACCG
      M.caprae  CGAGGAGTCCAACACCTTTGGGCTGCAGCTCGAGCTCACCG
M.tuberculosis  CGAGGAGTCCAACACCTTTGGGCTGCAGCTCGAGCTCACCG
```

Supplementary Figure S1. Alignment of *M. tuberculosis*, *M. caprae*, and *M. bovis*. These consensus sequences were taken from the hsp65-V15.2 in-house curated database.
